# GSDMD is associated with survival in human breast cancer but does not impact anti-tumor immunity in a mouse breast cancer model

**DOI:** 10.3389/fimmu.2024.1396777

**Published:** 2024-08-19

**Authors:** Bart Boersma, Viola Puddinu, Arnaud Huard, Sébastien Fauteux-Daniel, Pratyaksha Wirapati, Sofia Guedri, Jean-Christophe Tille, Thomas McKee, Mikael Pittet, Gaby Palmer, Carole Bourquin

**Affiliations:** ^1^ School of Pharmaceutical Sciences, University of Geneva, Geneva, Switzerland; ^2^ Institute of Pharmaceutical Sciences of Western Switzerland, University of Geneva, Geneva, Switzerland; ^3^ Department of Medicine, Faculty of Medicine, University of Geneva, Geneva, Switzerland; ^4^ Department of Pathology and Immunology, Faculty of Medicine, University of Geneva, Geneva, Switzerland; ^5^ Swiss Institute of Bioinformatics, Lausanne, Switzerland; ^6^ Division of Clinical Pathology, Geneva University Hospitals, Geneva, Switzerland; ^7^ Translational Research Centre in Onco-Hematology (CRTOH), Geneva, Switzerland; ^8^ AGORA Cancer Research Centre Lausanne, Lausanne, Switzerland; ^9^ Ludwig Institute for Cancer Research, Lausanne, Switzerland; ^10^ Geneva Centre for Inflammation Research, University of Geneva, Geneva, Switzerland; ^11^ Department of Anesthesiology, Pharmacology, Intensive Care and Emergencies, Faculty of Medicine, University of Geneva, Geneva, Switzerland

**Keywords:** cancer immunology, gasdermin D, breast cancer, Toll-like receptor 7, immunotherapy

## Abstract

Inflammation plays a pivotal role in cancer development, with chronic inflammation promoting tumor progression and treatment resistance, whereas acute inflammatory responses contribute to protective anti-tumor immunity. Gasdermin D (GSDMD) mediates the release of pro-inflammatory cytokines such as IL-1β. While the release of IL-1β is directly linked to the progression of several types of cancers, the role of GSDMD in cancer is less clear. In this study, we show that GSDMD expression is upregulated in human breast, kidney, liver, and prostate cancer. Higher *GSDMD* expression correlated with increased survival in primary breast invasive carcinoma (BRCA), but not in liver hepatocellular carcinoma (LIHC). In BRCA, but not in LIHC, high *GSDMD* expression correlated with a myeloid cell signature associated with improved prognosis. To further investigate the role of GSDMD in anticancer immunity, we induced breast cancer and hepatoma tumors in GSDMD-deficient mice. Contrary to our expectations, GSDMD deficiency had no effect on tumor growth, immune cell infiltration, or cytokine expression in the tumor microenvironment, except for *Cxcl10* upregulation in hepatoma tumors. *In vitro* and *in vivo* innate immune activation with TLR ligands, that prime inflammatory responses, revealed no significant difference between GSDMD-deficient and wild-type mice. These results suggest that the impact of GSDMD on anticancer immunity is dependent on the tumor type. They underscore the complex role of inflammatory pathways in cancer, emphasizing the need for further exploration into the multifaceted effects of GSDMD in various tumor microenvironments. As several pharmacological modulators of GSDMD are available, this may lead to novel strategies for combination therapy in cancer.

## Introduction

It is well established that inflammation plays an important role in cancer development. Chronic inflammation promotes tumor progression and resistance to treatment (1), whereas acute inflammatory responses can lead to protective anti-tumor immunity (2). An important mediator of inflammation is the cytokine IL-1β, which is produced in its immature form, pro-IL-1β, following activation of immune cells by a variety of stimuli, such as Toll-like receptor (TLR) ligands (3). Pro-IL-1β is cleaved by inflammatory caspases into its active form, IL-1β, after activation of a multimolecular complex called the inflammasome, and is then released into the extracellular space. Several mechanisms have been described for its release, one of which involves a form of cell death known as pyroptosis (4). The effector proteins of pyroptosis are gasdermin D (GSDMD) and other members of the gasdermin family (4). GSDMD can be cleaved by caspases 1, 4, 5 and 11 to release its active N-terminal domain. These fragments oligomerize to form pores in the cell membrane, through which IL-1β and other pro-inflammatory cytokines are released and that ultimately lead to cell death by pyroptosis (5).

The contrasting pro- and antitumoral roles of IL-1β are increasingly well recognized in cancer (3). In renal and breast carcinoma, several studies have shown that IL-1β drives a tumor-promoting transcriptional profile (6, 7). In addition, elevated levels of IL-1β expression correlate with p53 mutations, late-stage disease, and the basal-like subtype in breast cancer (7, 8). In a randomized, controlled trial, IL-1β inhibition decreased lung cancer incidence in a dose-dependent manner, although efforts to reproduce this finding were not successful to date (9). Further, IL-1β has been shown to enhance the recruitment of immunosuppressive myeloid cells to the tumor and to promote tumor angiogenesis (10, 11). On the other hand, IL-1β is essential for the generation of T cell memory, and adjuvants that promote production of mature IL-1β are more effective at inducing durable anticancer immunity (2).

The role of GSDMD and other members of the gasdermin family in cancer is less clear. Gasdermins A, B and E can induce pyroptosis in tumor cells, leading to improved anti-cancer immunity and control of tumor growth (12–14). GSDMD in contrast is predominantly expressed by myeloid cells in the tumor, including macrophages and dendritic cells (15). High intratumoral expression of GSDMD has been associated with poor prognosis in hepatocellular carcinoma (16), but this protein was found to be downregulated in colorectal cancer (CRC), and GSDMD deficiency enhanced the development of CRC in mice (17). How GSDMD in immune cells affects anticancer immune responses thus remains unclear.

In this study, we investigated the role of GSDMD in anticancer immunity. We show that *GSDMD* mRNA expression is upregulated in several types of human cancer and that this is associated with profound changes in the transcriptional profile of immune cell-associated genes in primary breast invasive carcinoma (BRCA) and liver hepatocellular carcinoma (LIHC). Using GSDMD-deficient mice, we found that growth of implanted breast cancer, known to be dependent on IL-1β, as well as of hepatoma tumors was independent of the expression of GSDMD in the tumor microenvironment. No changes were observed in immune cell infiltration or intratumoral cytokine expression in GSDMD-deficient, as compared to wild-type (WT) mice, with the exception of *Cxcl10* in hepatoma tumors. Upon activation of immune cells isolated from WT or *Gsdmd-/-* mice with pharmacological agents known to prime the inflammasome, such as TLR ligands, we did not observe any major difference in cytokine release or immune cell activation. In addition, when R848, a TLR7/8 agonist, was administered *in vivo*, no change in cytokine release or immune cell activation was observed in GSDMD-deficient, as compared to WT mice. Taken together, this data suggests that the role of GSDMD in cancer may vary according to the tumor type and is context-dependent.

## Results

### Higher GSDMD expression is associated with increased survival in patients with breast cancer, but not with hepatocellular carcinoma

**Figure 1:**
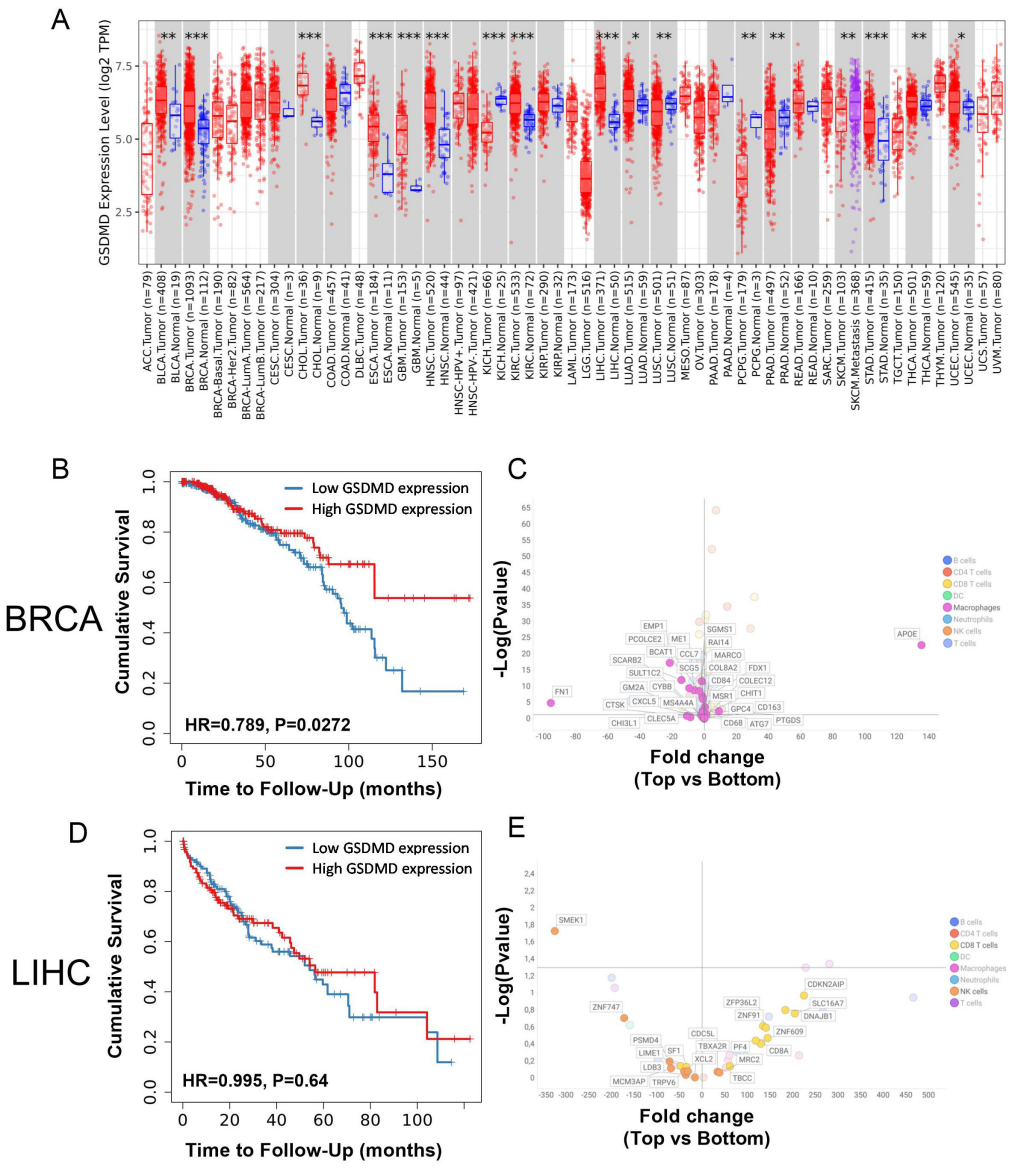
**(A)** Differential *GSDMD* gene expression (Log2) between tumor (red) and adjacent normal tissue (blue) from the TCGA database. **(B)** Probability of overall survival for patients with top 25% (red) vs. bottom 25% (blue) *GSDMD*-expressing BRCA patients. **(C)** Fold change in the expression of genes associated with immune cell types in top 25% vs. bottom 25% *GSDMD*-expressing BRCA patients. Fold change represent the ratio of top 25% over bottom 25% GSDMD-expressing patients. **(D)** Probability of overall survival for patients with top 25% (red) vs. bottom 25% (blue) *GSDMD*-expressing LIHC patients. **(E)** Fold change in the expression of genes associated with immune cell types in top 25% vs. bottom 25% *GSDMD* expressing LIHC patients. Fold change represent the ratio of top 25% over bottom 25% GSDMD expressing patients. *p<0.05; **p<0.01; ***p<0.001.

To determine whether gasdermin D (*GSDMD*) gene expression within the tumor was associated with differences in the anticancer immune response, the intratumoral expression of *GSDMD* in patients was correlated with clinical outcome and with immune cell infiltration using the TCGA repository. The expression of *GSDMD* mRNA in different types of cancer was first compared with its expression in the corresponding healthy tissues. *GSDMD* mRNA expression was upregulated in several types of cancer, including breast, kidney, liver, and prostate cancer (**Figure 1A**). The potential impact of intratumoral *GSDMD* expression on survival outcomes and immune cell-related genes was examined in, primary breast invasive carcinoma (BRCA) and liver hepatocellular carcinoma (LIHC). Higher *GSDMD* expression correlated with increased survival in BRCA, but not in LIHC patients (**Figures 1B, D**). A comparative analysis of the expression of genes associated with immune cell types was conducted in patients in the top and bottom 25% of *GSDMD* expression levels (18) (**Figures 1C, E**). For patients with BRCA, differences in *GSDMD* expression were mainly linked to differential expression patterns of macrophage-associated genes. Elevated *GSDMD* expression significantly correlated with decreased expression of markers such as *FN1*, *EMP1*, and *PCOLCE2* (**Figure 1C**, **Supplementary Table S1**), which are typically associated with M2-type macrophages and are indicative of a poor prognosis (19–21). This analysis did not reveal substantial differences in markers for other immune cell types in the context of BRCA. For LIHC patients, less expression of immune-related genes was detected and no clear myeloid signature was seen in LIHC patients with high *GSDMD* expression, in contrast to what was observed in BRCA patients. However, *GSDMD*-high patients exhibited an elevated expression of CD8 T cell-related genes, including *CDKN2AIP*, *DNAJB1*, and *SLC16A7*. Meanwhile, genes associated with NK cells, such as *SMEK1* and *YNF747*, were predominantly downregulated in this context (**Figure 1E**). In summary, high *GSDMD* expression was associated with improved survival in BRCA but not in LIHC patients. In addition, BRCA patients with high *GSDMD* expression showed a distinct downregulation of the M2-type macrophage signature, which was not the case for LIHC patients.

**Figure 2:**
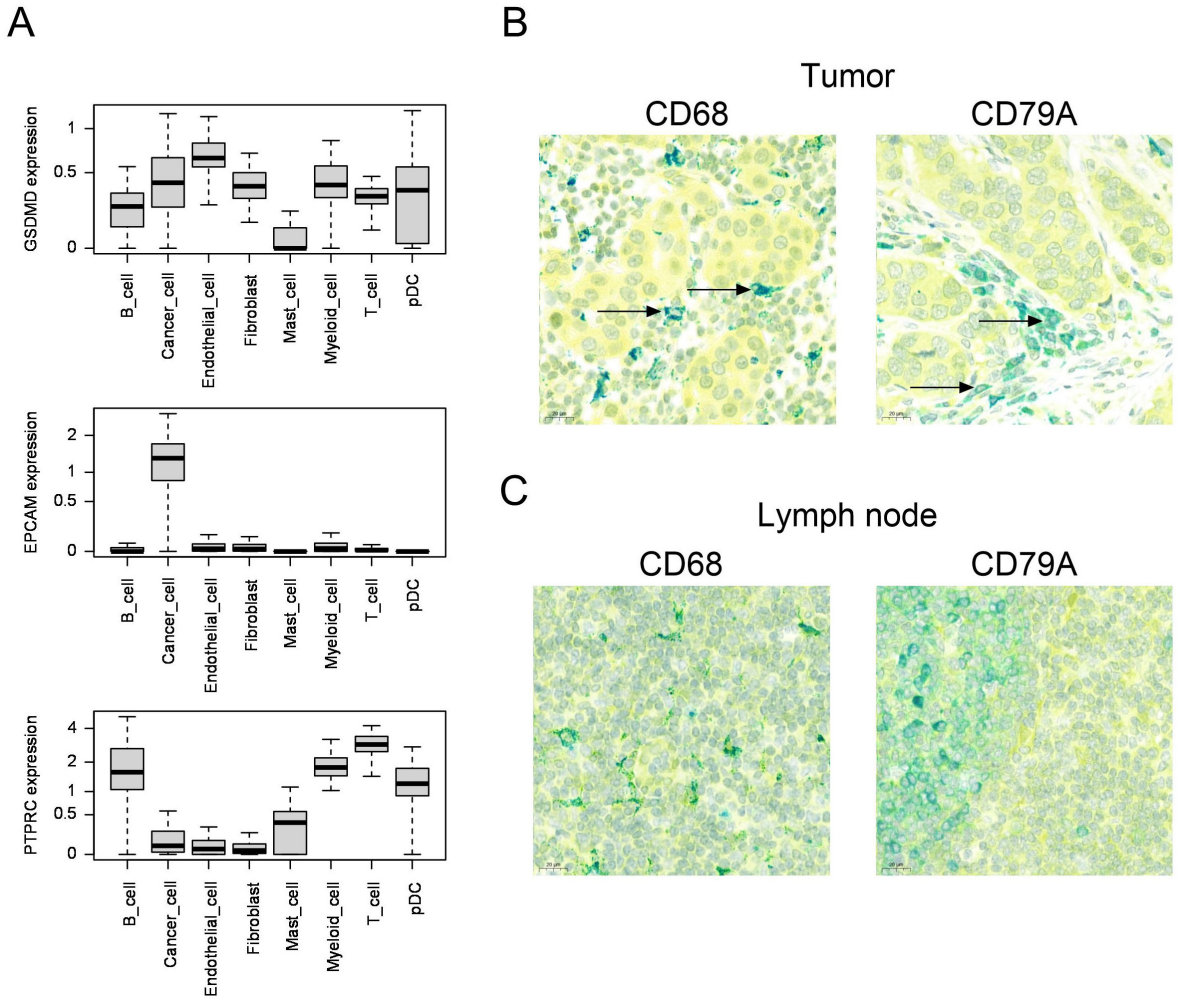
**(A)** Boxplots comparing the distribution of per-sample, per-cell type average expression of the *GSDMD*, *EPCAM* (epithelial control) and *PTPRC* (immune cell control) genes, in scRNA datasets from Bassez et al. (breast cancer, n=84)^22^. **(B, C)** Human breast cancer **(B)** or lymph node **(C)** samples co-stained for GSDMD (yellow) and CD68 (macrophage marker, teal color) or CD79A (B cell lineage marker, teal color) by immunohistochemistry. Colocalization is indicated by green color. The arrows show colocalization of GSDMD and CD68 or CD79A.

To determine which cell types expressed GSDMD, we used publicly available human single-cell RNA data sets from human breast cancer (n=84) (22) and liver cancer (n=21) (23). In breast cancer, we observed that *GSDMD* was expressed both in cancer cells and in the microenvironment, including endothelial cells, fibroblasts and most immune cell types, such as myeloid cells and B and T lymphocytes (**Figure 2A**). Expression of EpCAM and PTPRC (CD45) are shown as positive controls for cancer cells and immune cells, respectively. Average gene expression for individual samples is shown in **Supplementary Figure S1**. Similarly, in hepatocellular carcinoma, we observed expression of *GSDMD* in hepatocytes, endothelial cells, fibroblasts and different immune cell subsets (**Supplementary Figure S2**).

In order to confirm the expression of GSDMD at the protein level in breast cancer, we stained breast cancer samples for GSDMD by immunohistochemistry. In five patients with invasive ductal carcinoma, we found expression of GSDMD in tumor cells as well as in immune cells and endothelial cells (**Supplementary Figure S3**, **Table 1**). In all samples examined, GSDMD expression colocalized with the macrophage marker CD68 and the B-cell lineage marker CD79A (**Figure 2B**). A lymph node was used for control staining (**Figure 2C**).

**Table 1:** Expression of GSDMD in breast cancer.

Patient ID	Age	Sex	Tumor type	TNM stage	Grade	ER	PR	HER2	TIL	GSDMD staining		
										Tumor cells	Macro-phages	B cells
Patient 1	49	F	ductal	pT2 pN1a(sn)	G3	+	+	–	60%	+	+++	++
Patient 2	87	F	ductal	pT2 pN1a	G2	+	+	–	30%	+++	++	++
Patient 3	43	F	ductal	pT2 pN1a	G2	+	+	–	30%	+++	++	+++
Patient 4	47	F	ductal	pT2 pN1a	G3	+	+	–	50%	+	+++	+
Patient 5	61	F	ductal	pT1c pN0(sn)	G3	+	+	–	40%	++	+	++

Samples from six breast cancer patients were analyzed for GSDMD expression in tumor cells, macrophages (co-staining for CD68) and B lymphocytes (co-staining for CD79A) by immunohistochemistry.

Level of GSDMD staining in the indicated cell types is defined as + (low), ++ (medium) or +++ (high). Patient and tumor characteristics are indicated. ER: estrogen receptor; PR: progesterone receptor; HER2, human epidermal growth factor receptor 2; TIL, percentage of tumor-infiltrating lymphocytes within stroma-infiltrating leukocytes.

### Growth and immune cell infiltration is not impacted in syngeneic tumors implanted in GSDMD-deficient mice

**Figure 3:**
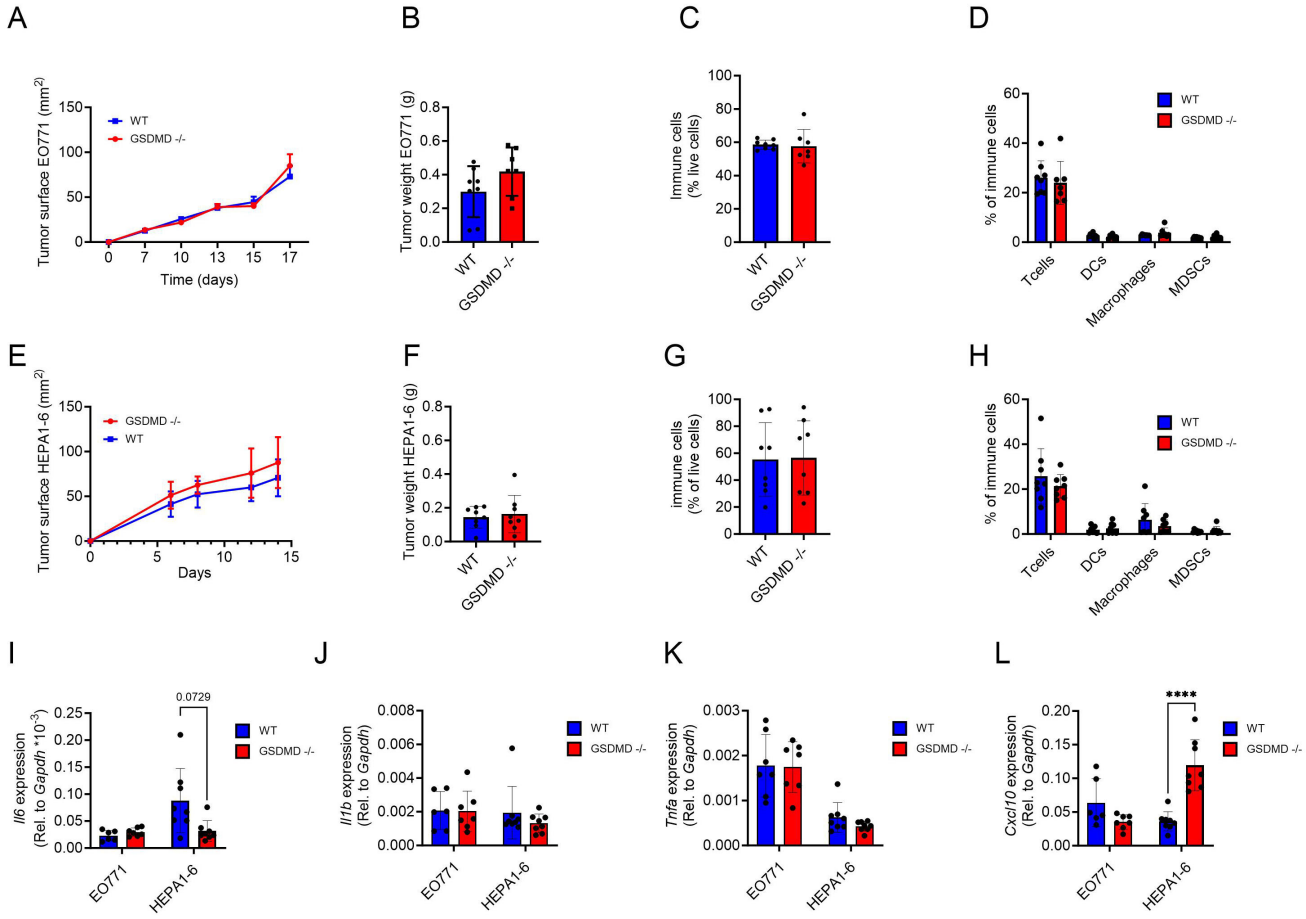
**(A–D)** EO771 tumors implanted in the mammary pad of WT (n=8) and *Gsdmd^-/-^* mice (n=7) were analyzed for tumor growth **(A)** and tumor weight **(B)**. Percentages of intra-tumoral immune cells (CD45.2^+^) **(C)** and percentages of T cells (CD3^+^), DCs (CD11b^+^, CD11c^+^), macrophages (CD11b^+^, F4/80^+^) and MDSCs (CD11b^+^, F4/80^+^, GR1^+^) in total immune cells **(D)** in EO771 tumors. **(E–H)** Hepa1-6 tumors implanted s.c. in WT (n=8) and *Gsdmd^-/-^* mice (n=8) were analyzed for tumor growth **(E)** and tumor weight **(F)**. Percentages of intra-tumoral immune cells **(G)** and percentages of T cells, DCs, macrophages and MDSCs in total immune cells **(H)** in Hepa1-6 tumors. **(I–L)** Gene expression of *Il-6* **(I)**, *Il-1β* **(J)**, *Tnf-α* **(K)** and *Cxcl10* **(L)** in whole EO771 and Hepa1-6 tumor samples from WT and *Gsdmd-/-* mice. Tumor growth graphs represent one experiment and each dot represent 1 mouse. In the qPCR data each dot represents the mean of a technical triplicate of each mouse. ****p<0.0001.

To specifically examine the role of GSDMD expression by host cells in the antitumor immune response, we used two different tumor models, one for breast cancer and one for hepatoma in GSDMD-deficient mice. The murine breast cancer cell line EO771 was selected as a breast cancer model, since the growth of EO771 tumors is dependent on IL-1β derived from the intra-tumoral myeloid compartment (24). First, to verify that myeloid cells from *Gsdmd* **^-/-^** mice did not release IL-1β upon classical stimulation of the inflammasome, bone marrow-derived macrophages (BMDM) from WT and *Gsdmd* **^-/-^** mice were stimulated using lipopolysaccharide (LPS) and adenosine triphosphate (ATP) (25). As expected, cells from WT, but not from *Gsdmd* **^-/-^** mice, secreted IL-1β following stimulation (**Supplementary Figure S4**). EO771 cells were then implanted in the mammary fat pad of WT and *Gsdmd* **^-/-^** mice, and tumor growth and immune cell infiltration were compared. No discernible differences in either tumor growth or tumor weight were observed between WT and *Gsdmd* **^-/-^** mice (**Figures 3A, B**). To gain further insights, the immune composition of the tumor was analyzed by flow cytometry. No significant variations in the overall immune cell infiltration within the tumor was observed between *Gsdmd* **^-/-^** and WT mice (**Figure 3C**). Furthermore, intratumoral T cell, DC, macrophage and MDSC percentages did not differ between the two experimental groups (**Figure 3D**). In addition, the cellular composition of the spleen and lymph nodes did not show any changes between tumor-bearing WT and *Gsdmd* **^-/-^** mice, nor did the proportion of CD4^+^ and CD8^+^ naïve, effector and memory T cells in tumor-draining lymph nodes (**Supplementary Figures S5**–**S7**).

A similar experiment was performed in a murine hepatoma model. Here, subcutaneous (s.c.) Hepa1-6 tumors were established in *Gsdmd* **^-/-^** and WT mice. Neither tumor growth nor tumor weight were influenced by the deficiency in GSDMD (**Figures 3E, F**). Moreover, intratumoral immune cell infiltration (**Figure 3G**), T cell, DC, macrophage and MDSC percentages showed no significant differences between WT and *Gsdmd* **^-/-^** mice (**Figure 3H**). The lack of GSDMD also had no significant impact on immune populations in the spleen of tumor-bearing mice or on the phenotype of CD4^+^ and CD8^+^ T cells in the tumor-draining lymph nodes (**Supplementary Figure S8**).

Subsequently, intratumoral gene expression levels for different cytokines and chemokines were assessed in EO771 and Hepa1-6 tumors. *Il6* mRNA expression levels were not significantly different between *Gsdmd* **^-/-^** and WT mice in either tumor type (**Figure 3I**). Unsurprisingly, as IL-1β production is regulated post-transcriptionally, *Il1b* mRNA expression levels were also unaffected (**Figure 3J**). *Tnfα* expression levels also did not show major differences (**Figure 3K**). In contrast, *Cxcl10* levels were significantly upregulated in mice lacking GSDMD in Hepa1-6 tumors, but not in EO771 tumors (**Figure 3L**). No significant difference was seen in the intratumoral expression levels of *Gzmb*, *Prf1*, *Il1r1*, *Tnfrsf1a*, *Ifng* and *Cxcl9* mRNA between *Gsdmd* **^-/-^** and WT mice in either tumor type (**Supplementary Figure S9**). In summary, the deficiency in GSDMD did not exert an impact on tumor growth, immune cell infiltration, or the immune landscape in either the EO771 breast cancer or the Hepa1-6 hepatoma model, although intratumoral *Cxcl10* levels were elevated in Hepa1-6 tumors grown in *Gsdmd^-/-^* mice.

### Immune cells from *Gsdmd* ^-/-^ mice respond to *in vitro* innate immune activation in a similar manner to cells from wild-type mice

**Figure 4:**
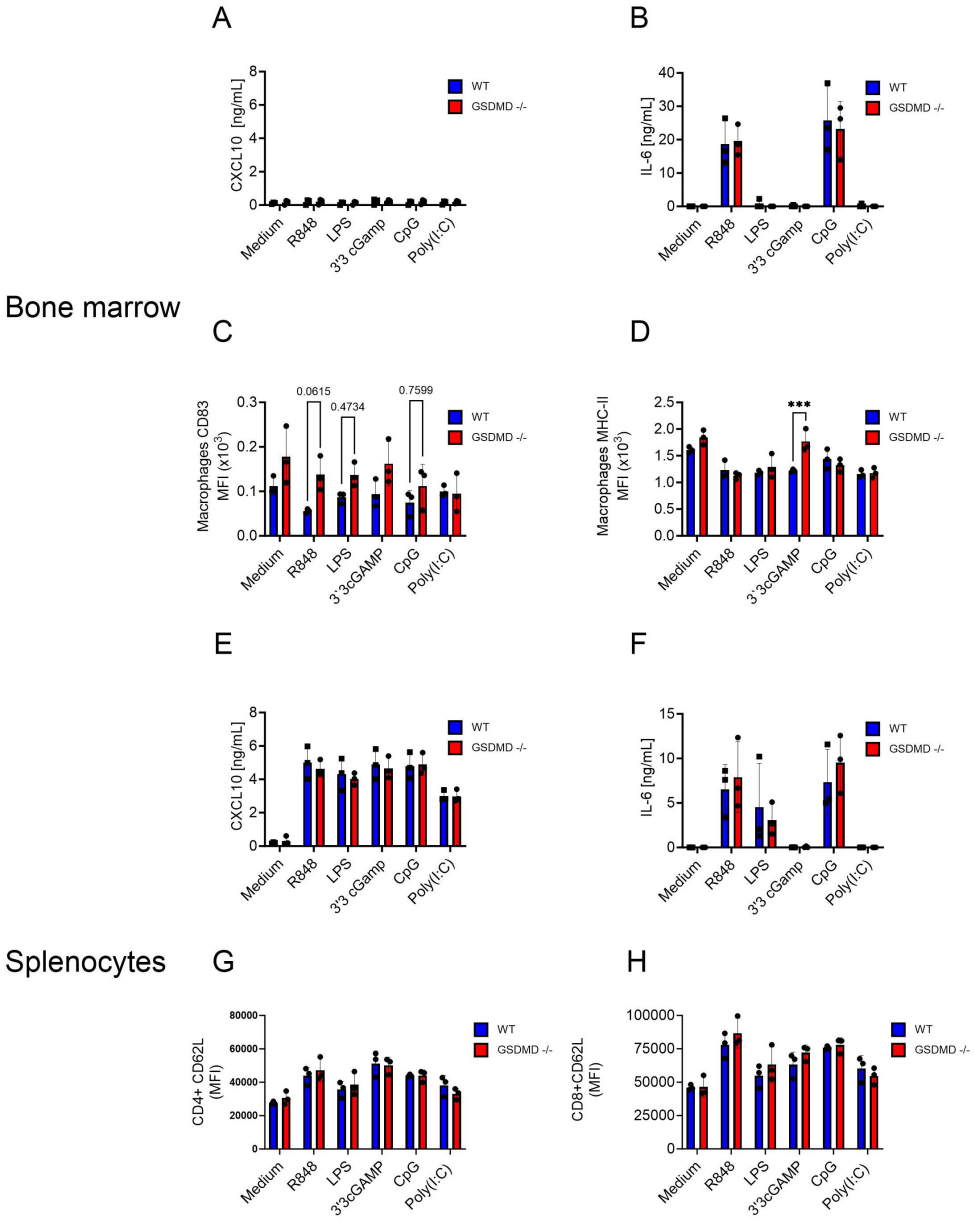
**(A-H)** Bone marrow **(A-D)** and splenocytes **(E-H)** from WT and *Gsdmd-/-* mice were stimulated with R848 (0.1 µg/mL), LPS (0.1 µg/mL), 3’3’cGAMP (10 µg/mL), CpG (30 µg/mL) and Poly(I:C) (200 µg/mL). CXCL10 **(A, E)** and IL-6 levels **(B, F)** in cell culture medium were measured 18h after stimulation. Cell surface CD83 **(C)** and MHC-II **(D)** levels were measured on macrophages in the bone marrow. Cell surface CD62L levels were measured on CD4^+^ **(G)** and CD8^+^ **(H)** T cells in the spleen. Each graph represents three experiments and each dot represents the mean of 3 biological triplicates.

The observed upregulation of *Cxcl10* mRNA expression in Hepa1-6 tumors in *Gsdmd* **^-/-^** mice suggested that GSDMD deficiency might influence the activation of immune cells. To examine this, splenocytes and bone marrow cells from *Gsdmd* **^-/-^** and WT mice were stimulated with activators for different innate immune pathways. Stimulants for Toll-like receptor (TLR) 7 (R848), TLR4 (LPS), STING (3’3’cGAMP), TLR9 (CpG) and TLR3 (polyI:C) were used. As indicators of immune cell activation, the levels of CXCL10 and IL-6 were measured in the cell culture medium. While CXCL10 was not induced in bone marrow cells, R848 and CpG induced IL-6 release, with no difference between *Gsdmd* **^-/-^** and WT cells (**Figures 4A, B**). Several activation markers were evaluated by flow cytometry on different immune cell subsets. For bone marrow cells, CD83 was not clearly upregulated on either macrophages or dendritic cells (DC) (**Figure 4C**, **Supplementary Figure S10A**). MHC-II was more highly expressed after STING activation on *Gsdmd* **^-/-^** macrophages than on WT macrophages, but this difference was not seen in DCs (**Figure 4D**, **Supplementary Figure S10B**). For splenocytes, CXCL10 release was induced by all stimulants and IL-6 release was induced by R848, LPS and CpG (**Figures 4E, F**). However, no difference between the two experimental groups was found. Within splenocytes, the activation markers CD62L and CD44 on CD4^+^ and CD8^+^ T cells and CD62L and CD69 on B cells were examined. CD62L showed an expected upregulation following stimulation with TLR ligands, but showed no difference between the two experimental groups on both CD4^+^ and CD8^+^ T cells (**Figure 4G, H**). The remaining activation markers on B cells, CD4^+^ and CD8^+^ T cells did also not show any significant changes between the two experimental groups (**Supplementary Figure S10C-F**). No IL-1β or free IL-18 were detected in the supernatants of stimulated bone marrow cells and splenocytes in these conditions (data not shown). Next, GSDMD cleavage was assessed by Western blotting upon LPS or R848 stimulation in combination with ATP in BMDM. Only the combination of LPS and ATP led to secretion of IL-1β and to GSDMD cleavage (**Supplementary Figure S11**). This differential sensitivity of inflammasome activation to LPS and R848 in BMDM is consistent with previous observations (26).

In conclusion, bone marrow and spleen cells isolated from *Gsdmd^-/-^* and WT mice showed similar responses in terms of CXCL10 and IL-6 release, as well as activation marker levels on different immune cell subsets, following *in vitro* stimulation with various innate immune pathway activators. Notably, despite the observed upregulation of *Cxcl10* in Hepa1-6 tumors in *Gsdmd^-/-^* mice, *in vitro* stimulation did not unveil substantial differences in immune cell activation between the two experimental groups, suggesting that the relationship between GSDMD deficiency and immune response activation might be better studied *in vivo.*

### *Gsdmd* ^-/-^ and WT mice show no difference in immune activation upon *in vivo* stimulation with a TLR7 ligand

**Figure 5:**
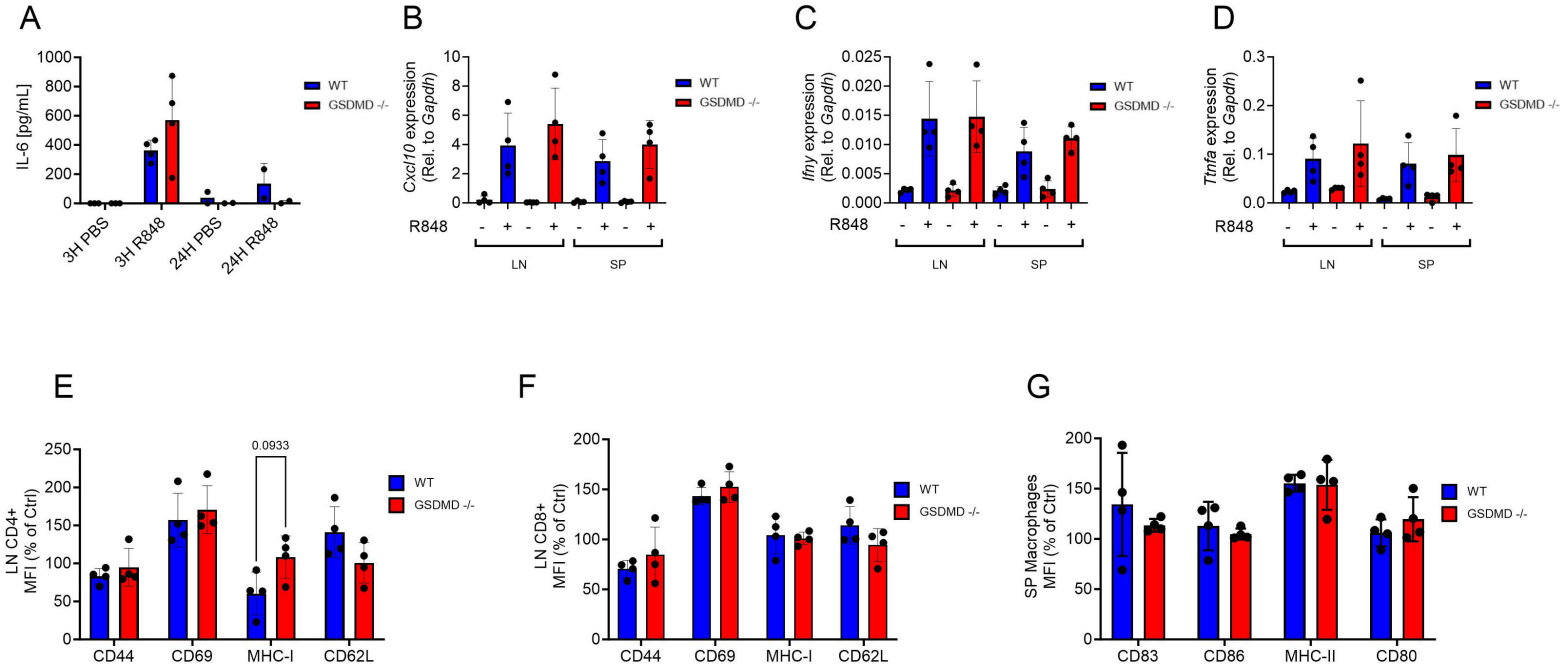
**(A)** Systemic IL-6 levels 3h and 24h after R848 (10 μg, s.c.) or PBS injection in *Gsdmd-/-* and WT mice (n=4 per group). **(B-D)** *Cxcl10* **(B)**, *Ifng* **(C)** and *Tnfa* **(D)** gene expression in the lymph nodes (LN) and spleen (SP) after 3h of R848 or PBS treatment. **(E, F)** Cell surface CD44, CD69, MHC-I and CD62L levels on CD4^+^ **(E)** and CD8^+^ **(F)** T cells (CD3^+^) from the lymph nodes after 3h of R848 treatment. **(G)** Cell surface CD83, CD86, MHC-II and CD80 levels on macrophages (CD11b^+^, F4/80^+^) from the spleen (SP) after 3h of R848 treatment. ELISA graphs represent one experiment and each dot represent 1 mouse. In the qPCR data each dot represents the mean of a technical triplicate of each mouse.

TLR7 stimulation in mice leads to the activation of myeloid cells that produce proinflammatory cytokines and chemokines, such as IL-6, CXCL10 and IFNγ. This in turn leads to the activation of T cells (27, 28). B cells express TLR7 and are also directly activated by TLR7 agonists (29, 30). To examine whether *Gsdmd* **^-/-^** mice responded differently to TLR7 activation than WT mice, mice were injected with the TLR7 ligand R848 or with PBS and serum cytokine levels were assessed after 3h and 24h. Serum IL-6 levels were increased as expected 3h after R848 injection, but no difference was seen between WT and *Gsdmd* **^-/-^** mice (**Figure 5A**). *Cxcl10* gene expression levels were increased in the lymph nodes and spleen 3h after R848 stimulation, but *Gsdmd* **^-/-^** mice exhibited no difference to WT mice (**Figure 5B**). A similar pattern was observed for *Ifng* and *Tnfa* gene expression in both the lymph nodes and spleen (**Figures 5C, D**). Immune cell activation was measured by flow cytometry in the lymph nodes, spleen and bone marrow of *Gsdmd* **^-/-^** and WT mice. The activation marker CD69 was increased on both CD4^+^ and CD8^+^ T cells in the lymph nodes 3h after R848 stimulation, but no difference was observed between experimental groups (**Figures 5E, F**). CD44, MHC-I and CD62L showed no increase at this time point upon stimulation in either group (**Figures 5E, F**). A similar pattern was observed on lymph node B cells and on CD4^+^ and CD8^+^ T lymphocytes and B cells in the spleen (**Supplementary Figures S14A-D**). In splenic macrophages, MHC-II levels increased following R848 stimulation, but no difference was seen between *Gsdmd* **^-/-^** and WT mice (**Figure 5G**). CD83, CD86 and CD80 only increased slightly following stimulation (**Figure 5G**). Findings were similar for splenic DCs (**Supplementary Figure S14E**). Considering these results, we did not test effects of TLR activation in tumor-bearing *Gsdmd* **^-/-^** and WT mice. In summary, *Gsdmd^-/-^* and WT mice responded similarly to TLR7 activation, showing no significant differences in serum cytokine levels or immune cell activation markers. The expected upregulation of *Il6*, *Cxcl10*, *Ifng*, and *Tnfa* gene expression after R848 stimulation was comparable between both groups, indicating that GSDMD deficiency did not markedly affect the response to TLR7 activation in the assessed tissues.

## Discussion

In this work, we explored how GSDMD influenced cancer growth and immune infiltration. Using a publicly available database, we have shown that *GSDMD* mRNA is overexpressed in different types of cancer, including breast cancer, bladder cancer, esophageal cancer, stomach cancer, glioblastoma, head and neck cancer, renal cell carcinoma, and hepatocellular carcinoma, supporting and extending previous findings in several types of cancer (31–33). Previous studies at protein level have suggested that in mice, GSDMD is mainly expressed in myeloid cells (15). Here we show that in human breast cancer and hepatocellular carcinoma, GSDMD is expressed in many immune cell subtypes and in tumor cells.

We then correlated GSDMD expression with survival in cancer patients. In breast cancer, we found that when comparing mRNA expression of GSDMD in the top 25% vs. bottom 25% *GSDMD*-expressing patients, high expression of *GSDMD* correlated with improved survival. This supports previous observations in a cohort of 108 patients with breast cancer, in which higher intratumoral protein levels of GSDMD correlated with lower pathological grade, smaller tumor size and lower TNM classification stage (34). We found that, in breast cancer, high *GSDMD* mRNA expression was associated with a clear myeloid cell signature, characterized by a downregulation of markers associated with M2-type macrophages and a poor prognosis (35). Others have reported that the secretion of IL-1β by tumor-infiltrating myeloid cells was associated with more advanced disease in primary breast cancer (7, 8). Since GSDMD is associated with better prognosis in this tumor type, these results suggest that in breast cancer, GSDMD may have other functions besides mediating the release of IL-1β.

In contrast to the findings in breast cancer, we found that enhanced expression of GSDMD in patients with hepatocellular carcinoma (HCC) did not correlate with improved overall survival, relapse-free survival or with a myeloid signature. This is in line with previous reports in patients suffering from HCC that used in part the same source data (16, 36, 37). In a cohort of 50 HCC patients, GSDMD was upregulated at the mRNA and protein levels compared to adjacent normal tissue and the cleaved N-terminal GSDMD was also elevated in HCC samples (16). Upregulation of GSDMD in these patients correlated with microvascular invasion, poor differentiation, and higher TNM classification (16). The positive or negative association of elevated GSDMD expression with survival differs according to the tumor type studied: in urothelial carcinoma and melanoma, high GSDMD expression was found to correlate with an increase in overall survival, as observed for breast cancer in this study (38). In addition, high expression of genes associated with pyroptosis was associated with improved prognosis in breast cancer (39). In contrast, elevated GSDMD expression correlated with worse prognosis in adrenal cortex carcinoma, low grade glioma, renal cancer and uveal melanoma (15, 38). Thus, intratumoral GSDMD expression may differently impact antitumor immunity depending on the tumor type and context.

The induction of pyroptosis in tumor cells by gasdermins has clearly been shown to promote anti-tumor immunity (12, 13). To assess the impact of GSDMD in the tumor microenvironment rather than tumor cell-intrinsic GSDMD, we implanted EO771 breast cancer cells into the mammary fat pads of GSDMD-deficient mice. No difference in tumor growth or immune cell infiltration was observed compared to WT mice. Interestingly, previous studies have shown that EO771 tumors are dependent on IL-1β derived from the myeloid compartment in the tumor, which drives tumor progression, and that treatment with an IL-1 receptor antagonist inhibited tumor growth (24, 40). The role of the inflammasome itself in EO771 tumors is however controversial, as in one study EO771 tumor growth was reduced in mice deficient for essential components of the inflammasome, caspase 1 or NLRP3 (24), whereas in another study tumor growth was independent of NLRP3 and of GSDMD, as seen in our study (40). We also found no difference in tumor growth in GSDMD-deficient mice bearing Hepa1-6 hepatoma tumors, in contrast to a model of hepatocarcinogenesis in which GSDMD-deficient mice exhibited smaller tumors (16). As seen in patients, the role of GSDMD in mouse models of cancer seems to depend on the tumor type and the context. It is possible that environmental factors, such as microbiota composition may influence the role of GSDMD, as was suggested previously for the NLRP3 inflammasome (41).

We did not observe differences in the composition of immune infiltrates between GSDMD-deficient and WT mice in either EO771 or Hepa1-6 tumors. We further did not observe differences in the intratumoral expression of proinflammatory cytokines, such as IL-1β, IL-6 or TNFα. However, the chemokine CXCL10 was upregulated in Hepa1-6 tumors in GSDMD-deficient mice but was not associated with a reduction in tumor growth or a difference in immune cell infiltration. This chemokine is induced by type I interferon, which in cancer is produced upon activation of the STING-cGAS pathway (2). Interestingly, GSDMD suppresses the cGAS-driven type I interferon response by depleting intracellular K^+^, and can, in this manner, promote tumor progression (15, 42). It is thus possible that this mechanism supported the increase in *Cxcl10* expression in these tumors. Since TLR ligands play an important role in the priming of the inflammasome machinery, we further explored the interaction between pattern-recognition receptors and GSDMD by activating immune cells from WT and GSDMD-deficient mice with ligands for Toll-like receptors and for the STING pathway. We found generally no difference in innate immune activation, except for MHC-II expression on macrophages following STING activation by cGAMP. Further, no difference was detected following *in vivo* stimulation of WT and GSDMD-deficient mice with a TLR7 ligand, that has been shown to induce IL-1β release in a GSDMD-dependent manner (43). Clearly, GSDMD is dispensable for the effects demonstrated. Although this model does not indicate whether the pyroptosis cascade is activated under these conditions, the absence of effect may also be due to the fact that some functions of GSDMD may be redundant with other members of the gasdermin family (5). In the light of the promising therapeutic applications of TLR7/8 agonists and their potential for combination immunotherapies (28), these findings may have important clinical implications.

In addition to its well-described role for the release of pro-inflammatory cytokines and pyroptosis, and the suppression of the STING-cGAS pathway, GSDMD may impact anticancer immunity by other mechanisms. In the context of liver carcinoma, GSDMD upregulates PD-L1 expression through phosphorylation of STAT1 (16). We however did not observe any decrease in PD-L1 expression in EO771 or Hepa1-6 tumors in GSDMD-deficient mice (data not shown). In a melanoma model, GSDMD deficiency enhanced the response to anti-PD-L1 treatment, whereas no difference in tumor growth was seen in GSDMD-deficient mice in the absence of treatment (15). It is therefore possible that under certain conditions, the absence of GSDMD only results in improved anti-tumor immunity in combination with immunotherapy. Thus, the combination of a GSDMD inhibitor and a checkpoint inhibitor may provide a promising strategy in selected patients (15).

In summary, although GSDMD expression was associated with survival and a decreased M2-type macrophage signature in patients with breast cancer, we did not observe a major impact of GSDMD deficiency in two different mouse cancer models, of which one is known to be IL-1β-dependent. These findings were somewhat unexpected, considering the clearly described role of GSDMD for the release of IL-1β and the well-known impact of inflammation on cancer development and progression. Furthermore, although TLR ligands play an important role for the priming of the inflammasome machinery and represent a promising strategy in cancer immunotherapy, we showed that GSDMD deficiency had only a minor effect on immune activation by TLR ligands *in vitro* and *in vivo*. However, additional studies with other inflammasome stimuli are needed to determine the full repertoire of GSDMD functions in tumor-immune relationships. Our findings suggest that the role of GSDMD in cancer is strongly context-dependent. In view of the emerging novel molecular mechanisms associated with GSDMD function, such as its interplay with mitochondria and its regulation by posttranslational modifications (44), these results highlight the need for further investigation into the multiple activities of GSDMD in different tumor environments. As several pharmacological modulators of GSDMD are available, this may lead to novel strategies for combination therapy in cancer.

## Materials and methods

### Bulk RNAseq analysis

TIMER2.0 (*timer.cistrome.org*) was used to analyse bulk tumor transcriptome data generated by the TCGA Research Network: https://www.cancer.gov/tcga. The statistical significance of *GSDMD* expression differences between healthy tissue and tumor tissue was computed by the Wilcoxon test using TIMER2.0. An adjusted partial Spearman’s test was used to calculate a rho value as the degree of gene correlation. Patients with the highest (top 25%) and lowest (bottom 25%) *GSDMD* expression were segregated into two cohorts, in which overall survival was compared with a Cox proportional-hazards model. A Wald test was used to determine the P value. MRNA expression data for BRCA and LIHC cancer types was downloaded from the TCGA portal. The signature genes previously described (18) were extracted from the expression data for the top 25% and bottom 25% GSDMD expressing patient cohorts and differential expression was visualized using Tibco Spotfire 12.5. For comparison the two-tailed t test was used.

### Single cell RNA seq analysis

To show the expression of *GSDMD* according to cell types, we used publicly available scRNA datasets from breast cancer (n = 84) (22) and hepatocellular carcinoma (n = 21) (23). The original cell type annotation from the respective authors was used. Pseudo-bulk summaries per patient were constructed by averaging the gene expression over all cells in a given cell type and plotted using the method of Bill et al. (2023) (45). Epithelial cell adhesion molecule (EpCAM) and protein tyrosine phosphatase receptor type C (PTPRC), also known as CD45, were used as controls to show specificity of the patterns of expression in epithelial and immune cells, respectively, under this analysis approach.

### Immunohistochemistry

Immunohistochemical multiplex staining was performed on 4-μm sections from FFPE blocks using the fully automated Discovery Ultra system (ROCHE Diagnostics). The process included baking, deparaffinization, cell conditioning, staining, IHC, counterstaining, and titration. Antigen retrieval was conducted using CC1 for 64 minutes, followed by incubation with a 2% normal goat serum solution at 37°C for 32 minutes. A peroxidase blocking solution was then applied.

For single stainings, rabbit polyclonal anti-GSDMD antibody (20770-1-AP, Proteintech) was used at a 1:200 dilution and incubated for 60 minutes at 37°C. The primary antibody was detected with anti-rabbit HRP complex and revealed by diaminobenzidine using automated routine procedures. Brown coloration corresponds to a positive staining.

For double stainings, the primary antibody was followed by an anti-rabbit-AP system for 16 minutes and a Yellow detection kit. Antibody denaturation was performed using CC2 at 100°C for 24 minutes before the second antibody incubation. For the second marker, mouse monoclonal anti-CD79a antibody (M7050, DAKO) or mouse monoclonal anti-CD68 PG antibody (M0876, DAKO) were used at a 1:50 dilution and incubated for 60 minutes at 37°C. An amplification system was then applied, followed by OmniMap Mouse HRP for 24 minutes, and Teal detection reagents for 32 minutes. Hematoxylin and Bluing Reagent were used as counterstains.

### Generation of bone marrow-derived macrophages and NLRP3 stimulation

Bone marrow cells were flushed out of freshly isolated tibias and femurs of 8-12 week-old male or female C57BL/6N-Gsdmd^em4Fcw^/J (*Gsdmd^-/-^*; The Jackson Laboratory, 032410) or WT C57BL/6N mice, using PBS. The cell suspension was flushed through a 40 µM filter (Greiner, 7542041). Red blood cells were lysed using BD Pharm Lyse™ Lysing Buffer (BD, 555899). Cells were then cultured in differentiation medium composed of RPMI (GIBCO, 11875093), (Pan Biotech, p30-3300), 1% Penicillin-Streptomycin (Gibco, 15140122), 1% L-glutamine (GIBCO, 25030081), 0.5% sodium pyruvate (GIBCO, 11360070), 0.1% β-mercaptoethanol (GIBCO, 21985023), and M-CSF (10ng/ml, Miltenyi Biotec, 130-101-705). BMDM were cultured at a density of 80.000 cells/cm^2^ in a 37°C, 5% CO_2_ humidified environment. Medium was refreshed 2 days and 4 days after isolation. On day 6, BMDM were collected by scraping in PBS, seeded at 6.25x10^5^ cells/cm^2^ and left to adhere overnight in a 37°C, 5% CO_2_ humidified environment. Cells were stimulated with lipopolysaccharide (LPS; 1 ug/ml, Invivogen, tlrl-eblps) for 3 hours, before adenosine triphosphate (ATP; 10 mM, Sigma, A2383) in Opti-MEM™ (Gibco, 31985062) was added for 1 hour. Culture supernatants were collected for measurements of IL-1β levels. IL-1β levels were measured using ELISA MAX™ following manufacturer’s instructions.

### *In vivo* injection of tumor cells

EO771 (ATCC CRL-3461) and Hepa1-6 (ATCC CRL-1830) murine cell lines were cultured using respectively DMEM (Gibco, 41965-039), 10% FBS (Pan Biotech, p30-3300), 1% Penicillin-Streptomycin (Gibco, 15140122) or DMEM (GIBCO, 41965-039), 10% FBS (Pan Biotech, p30-3300) medium in a 37°C, 5% CO_2_ humidified environment. Cells were detached using trypsin-EDTA (0.5%) (Gibco, 15400054). Cell suspensions were washed twice using PBS (Gibco, 10010015). Hepa1-6 cells were then injected at a concentration of 5x10^6^ cells/mL in a volume of 200 µL s.c. in the right upper flank of 8-12 week-old female *Gsdmd^-/-^* or WT C57BL/6N mice. For EO771 cell injection, mice were anesthetized using isofluorane (Piranal, B03A16C). EO771 cells were injected into the 4^th^ mammary pad of 8-12 week-old female *Gsdmd^-/-^* or WT C57BL/6N mice at a concentration of 0.5x10^6^ cells/mL in a volume of 20 µL. Tumor surface was calculated by multiplying the width and length of the tumor. Mice were sacrificed by CO_2_ euthanasia (Tem Sega automate) when the tumor surface reached 200 mm^2^. For mice bearing Hepa1-6 tumors, the tumor, spleen and the two axillary lymph nodes were collected. For mice bearing EO771 tumors, the tumor, spleen and the two subiliac lymph nodes were collected. Tissue was stored in TRIzol™ (ThermoFisher, 15596026) for RNA isolation. For flow cytometry, tumors were digested using a Tumor Dissociation Kit (130-096-730, Miltenyi) and the gentleMACS™ (130-096-427, Miltenyi) 37C_m_TDK_2 program. The digested tumors, spleen and lymph nodes were passed through a 40 µM filter (Greiner, 7542041) and red blood cells were lysed using BD Pharm Lyse™ Lysing Buffer (BD, 555899). All animal studies were approved by the Geneva cantonal authority for animal experimentation. Homozygous *Gsdmd^-/-^* and WT C57BL/6N mice for all mentioned *in vivo* experiments were bred in house and maintained in the SPF facility of the Geneva University School of Medicine (Geneva, Switzerland).

### Immune cell stimulation with TLR ligands

Bone marrow cells (2x10^6^ cells/mL) and splenocytes (2x10^6^ cells/mL) were seeded in a 200 µL volume in a 96-well plate (Greiner, 655083) and left overnight in a 37°C, 5% CO_2_ humidified environment. All cells were cultured in differentiation medium without M-CSF. The cells were stimulated using R848 (0.1 µg/mL, InvivoGen, tlrl-r848), LPS (0.1 µg/mL, Invivogen, tlrl-3pelps), 3’3’cGAMP (10 µg/mL, InvivoGen, tlrl-nacga), CpG (30 µg/mL, InvivoGen, ODN 1585) or poly(I:C) (200 µg/mL, InvivoGen, tlrl-picw). After an 18-hour incubation, the plate was centrifuged at 400 g for 5 min and conditioned medium was collected. IL-6 levels were measured using ELISA MAX™. CXCL10 was measured using a DuoSet ELISA (R&D systems, DY466). Activation marker expression was assessed by flow cytometry.

### Western blot

The samples were separated using SDS-PAGE and then transferred onto a membrane (Macherey-Nagel). The membrane was blocked in 0.05% Tris-buffered saline, Tween 20, containing 5% milk for 1 hour at room temperature and immunoblotting was performed with anti-GSDMD antibody (Abcam, EPR19828). Immunoreactive bands were visualized by Odyssey (LICOR) using appropriate secondary reagents.

### *In vivo* R848 stimulation

For the *in vivo* R848 stimulation, R848 (invivogen, TLR-R848) was brought to a concentration of 2 µg/mL in PBS and 200 µl were injected s.c. into 8-12 week-old female *Gsdmd^-/-^* or WT C57BL/6N mice. Mice were sacrificed after 3 or 24 hours and lymph nodes, blood, spleen, bone marrow and liver were collected. Tissues were prepared for RNA isolation or for flow cytometry as described above. IL-6 levels were measured in serum using ELISA MAX™ (Biolegend, 431316), as this cytokine is highly sensitive to measure activity of TLR7 ligands (46, 47).

### Quantitative real-time PCR analysis for gene expression

Total RNA was extracted using Trizol (Thermo Fischer). cDNA was generated from 1 µg total RNA using a High Capacity cDNA Reverse Transcription kit (Thermo Fischer, 4368814). Non-reverse-transcribed RNA samples were used as negative controls. PCR reactions were executed on a QuantStudio 5 system, using PowerUp SYBR Green Master mix (Thermo Fisher, A25742) and the primers listed in **Table 2**.

**Table 2:** List of primers used and corresponding sequences.

Target	Sequence 5’-3’
*Il6* fw	GTCCTTCCTACCCCAATTTC
*Il6* rev	GCCGAGTAGATCTCAAAGTG
*Tnfa* fw	AAATGGCCTCCCTCTCAT
*Tnfa* rev	CCTCCACTTGGTGGTTTG
*Il1b* fw	GTGTCTTTCCCGTGGACCTT
*Il1b* rev	AATGGGAACGTCACACACCA
*Cxcl10* fw	GCCGTCATTTTCTG CTCAT
*Cxcl10* rev	GCT TCCCTATGGCCCTCATT
*Gzmb* fw	CTGCTCACTGTGAAGGAAGTATAA
*Gzmb* rev	AGCTCTAGTCCTCTTGGCCT
*Prf1* fw	GAGAAGACCTATCAGGACCA
*Prf1* rev	AGCCTGTGGTAAGCATG
*Ifng* fw	AGGAACTGGCAAAAGGATGG
*Ifng* rev	ATGTTGTTGCTGATGGCCTG
*Il1r1* fw	GCACGCCCAGGAGAATATGA
*Il1r1* rev	AGAGGACACTTGCGAATATCAA
*Tnfrsf1a* fw	GCCTGCTGCTGTCACTGGTGCTCCT
*Tnfrsf1a* rev	AGTCCTGGGGGTTTGTGACATTTGC
*Cxcl9* fw	GTTCGAGGAACCCTAGTGAT
*Cxcl9* rev	GCTTGGGGCAAACTGTTTGA

### Flow cytometry

Cell suspensions were washed with PBS and treated with Fc Receptor block (1:200, TruStain FCX anti-mouse CD16/32, Biolegend, S17011E) in FACS buffer (PBS, 0.5% BSA (PAN Biotech, P06-1391500) and 2mM EDTA (Promega, V4231)). After 15 min of blocking, antibodies in FACS buffer were added to stain the cells. Antibodies and dilutions used for the staining are listed in **Table 3**. Marker expression was assessed using a Novocyte Flow Cytometer 3000 (Agilent). Flow cytometry data was analysed using FlowJo 10. Gating strategies used are outlined in **Supplementary Figures S4**, **S5**, **S8** and **S9**.

**Table 3:** List of flow cytometry reagents used.

Target	Color	Catalog #	Supplier	Dilution
CD45.2	APC-Cy7	109824	Biolegend	1:200
CD3	APC	100236	Biolegend	1:200
CD19	PE-Dazzle	115554	Biolegend	1:400
CD4	BV510	100553	Biolegend	1:400
CD8	BV785	100749	Biolegend	1:400
CD11b	PercP	101230	Biolegend	1:200
CD11c	BV605	117333	Biolegend	1:200
F4/80	FITC	123101	Biolegend	1:400
GR1	BV570	108431	Biolegend	1:200
Zombie	Pacific Blue	423113	Biolegend	1:1000
CD3	PercP	100218	Biolegend	1:200
CD4	FITC	100406	Biolegend	1:400
CD8	PE-Cy7	100722	Biolegend	1:400
cd11b	APC	101212	Biolegend	1:200
CD44	BV605	103047	Biolegend	1:200
CD62L	PE	104408	Biolegend	1:200
CD69	BV510	104532	Biolegend	1:200
CD11b	BV570	101233	Biolegend	1:200
CD11c	PE-Cy7	117318	Biolegend	1:200
MHC-II	BV650	107641	Biolegend	1:200
CD83	APC	121510	Biolegend	1:200

### Statistics

Data represent the average and SD of individual experiments or mice. A 2-way ANOVA, using Graphpad Prism 10, was used to determine P values. (GraphPad Software, La Jolla, CA, USA; *p<0.05; **p<0.01; ***p<0.001; ****p<0.0001).

## Data Availability

The original contributions presented in the study are included in the article/**Supplementary Material**. Further inquiries can be directed to the corresponding author.

## Supplementary material

Supplementary Figure 1 Scatter plots showing the average expression of the genes GSDMD, EPCAM and PTPRC by tumor samples (individual dots) and cell types, based on scRNA dataset from Bassez et al. (2021) (breast cancer, n=84).

Supplementary Figure 2
**(A)** Boxplots comparing the distribution of per-sample, per-cell type average expression of the genes GSDMD, EPCAM (epithelial control) and PTPRC (immune cell control), in scRNA datasets from Lu et al. (2022) (hepatocellular carcinoma, n=21). **(B)** Scatter plots showing the average expression of the genes GSDMD, EPCAM and PTPRC by tumor samples (individual dots) and cell types, based on scRNA dataset from Lu et al. (2022) (hepatocellular carcinoma, n=21).

Supplementary Figure 3
**(A–F)** Human breast cancer samples stained for GSDMD (brown) by immunohistochemistry.

Supplementary Figure 4 IL-1β release from BMDM of WT (blue) and *Gsdmd^-/-^* (red) mice after 3h LPS (100ng/mL) followed by 1h ATP (5mM) treatment.

Supplementary Figure 5
**(A–G)** Flow cytometric analysis of immune cells in tumor **(A)**, spleen **(B–D)** and lymph nodes (LN) **(E–G)** of EO771 tumor-bearing WT and Gsdmd-/- mice. **(A)** Percentages of CD4+ and CD8+ T cells (CD3 +) and B cells (CD19 +) in the tumor. Percentages of **(B)** CD45 + cells, **(C)** myeloid cells (CD11b +), DCs (CD11b +, CD11c +), macrophages (CD11b +, F4/80 +), and MDSCs (CD11b +, F4/80 +, GR1 +), and **(D)** total T cells (CD3+), CD4 + T cells, CD8 + T cells and B cells **(D)** in the spleen of EO771 tumor-bearing WT and Gsdmd-/- mice. **(E)** Percentages of total T cells (CD3+), CD4 + T cells and CD8 + T cells in the draining lymph nodes (LN) of EO771 tumor-bearing WT and *Gsdmd^-/-^* mice. Percentages of effector (CD44 +), memory (CD44 +, CD62L +) and naïve (CD62L +) CD4 + T cells (F) and CD8 + T cells (G) in draining lymph nodes of EO771 tumor-bearing WT and *Gsdmd^-/-^* mice.

Supplementary Figure 6 Gating strategy for the analysis of EO771 and Hepa1-6 tumors and spleens of healthy or tumor bearing WT and Gsdmd-/- mice. The first gate was set to exclude cell debris. The second gate was used to select single cells. Then dead cells were excluded with the help of a viability marker. Using CD45, the fourth gate was used to select immune cells. Then different subsets of immune cells were defined as follows: CD19+, CD3+CD8+, CD3+CD4+, CD11b+CD11c+, CD11b+F4/80+GR1-, CD11b+F4/80-GR1+.

Supplementary Figure 7 Gating strategy for the analysis of LN of EO771 and Hepa1-6 tumor bearing WT and Gsdmd-/- mice. The first gate was set to exclude cell debris. The second gate was used to select single cells. Then dead cells were excluded with the help of a viability marker. Using CD45, the fourth gate was used to select immune cells. Then different subsets of immune cell subsets were defined as follows: CD3+CD8+, CD3+CD8+CD44+CD62L+, CD3+CD8+CD44- CD62L+, CD3+CD8+CD44+CD62L-, CD3+CD4+, CD3+CD4+CD44+CD62L+, CD3+CD4+ CD44-CD62L+, CD3+CD4+CD44+CD62L-.

Supplementary Figure 8
**(A–G)** Flow cytometric analysis of immune cells in tumor **(A)**, spleen **(B–D)** and lymph nodes (LN) **(E–G)** of Hepa1-6 tumor bearing WT and Gsdmd-/- mice. **(A)** Percentages of CD4+ and CD8+ T cells (CD3 +) and B cells (CD19 +) in the tumor. Percentages of **(B)** CD45 + cells, **(C)** myeloid cells (CD11b +), DCs (CD11b +, CD11c +), macrophages (CD11b +, F4/80 +), and MDSCs (CD11b +, F4/80 +, GR1 +), and **(D)** total T cells (CD3+), CD4 + T cells, CD8 + T cells and B cells **(D)** in the spleen of Hepa1-6 tumor-bearing WT and Gsdmd-/- mice. (**(E)** Percentages of total T cells (CD3+), CD4 + T cells and CD8 + T cells in the draining lymph nodes (LN) of Hepa1-6 tumor-bearing WT and Gsdmd-/- mice. Percentages of effector (CD44 +), memory (CD44 +, CD62L +) and naïve (CD62L +) CD4 + T cells **(F)** and CD8 + T cells **(G)** in draining lymph nodes of Hepa1-6 tumor-bearing WT and Gsdmd-/ mice.

Supplementary Figure 9
**(A–F)** Gene expression of Gzmb **(A)**, Prf1 **(B)**, Il1r1 **(C)**, Tnfrsf1 **(D)**, Ifny **(E)** and Cxcl9 **(F)** in whole EO771 and Hepa1-6 tumor samples from WT and Gsdmd-/- mice.

Supplementary Figure 10
**(A–F)** Bone marrow cells **(A, B)** and splenocytes **(C–F)** of WT and Gsdmd-/- mice were stimulated with R848 (0.1 μg/mL), LPS (0.1 μg/mL), 3’3’cGAMP (10 μg/mL), CpG (30 μg/mL) and Poly(I:C) (200 μg/mL). Cell surface levels of activation markers were measured 18 hours after stimulation. Cell surface CD83 **(A)** and MHC-II **(B)** levels on bone marrow DCs (CD11b+CD11c+). Cell surface CD62L **(C)** and CD69 **(D)** levels on spleen B cells (CD19+). Cell surface CD44 levels on spleen CD4 + T cells (CD3 +) **(E)** and CD8 + T cells (CD3 +) **(F)**.

Supplementary Figure 11 IL-1β **(A)** GSDMD **(B)** levels from BMDM after 3h LPS (100ng/mL) or R848 (0.1 μg/mL) followed by 1h ATP (5mM) treatment.

Supplementary Figure 12 Gating strategy for the analysis of stimulated splenocytes isolated from WT and Gsdmd-/- mice. The first gate was set to exclude cell debris. The second gate was used to select single cells. Then dead cells were excluded with the help of a viability marker. Using CD45, the fourth gate was used to select immune cells. Then different subsets of immune cell subsets were defined as follows: CD19+, CD3+CD8+, CD3+CD4+.

Supplementary Figure 13 Gating strategy for the analysis of stimulated bone marrow isolated from WT and Gsdmd-/- mice. The first gate was set to exclude cell debris. The second gate was used to select single cells. . Then dead cells were excluded with the help of a viability marker. Using CD45, the fourth gate was used to select immune cells. Then different subsets of immune cell subsets were defined as follows: CD11b+CD11c+ and CD11b+F4/80+.

Supplementary Figure 14
**(A–E)** flow cytometric analysis of immune cells in lymph nodes **(A)** and spleen **(B–E)** 3H after R848 (10 μg, s.c.) or PBS injection in WT and Gsdmd-/- mice. **(A)** Cell surface CD44, CD69, MHC-I and CD62L levels in lymph node B cells (CD19+). **(B)** Cell surface CD83, CD86, MHCII and CD80 levels in spleenCD4+ T cells (CD3+) **(C)**, CD8+ T cells (CD3+), **(D)** B cells (CD19+) and **(E)** DCs (CD11b+, CD11c+).
