# Supplementary figures and images for: GSDMD is associated with survival in human breast cancer but does not impact anti-tumor immunity in a mouse breast cancer model
Source: Front Immunol. 2024 Aug 19;15:1396777. doi: 10.3389/fimmu.2024.1396777 (PMC11366651; doi:10.3389/fimmu.2024.1396777)

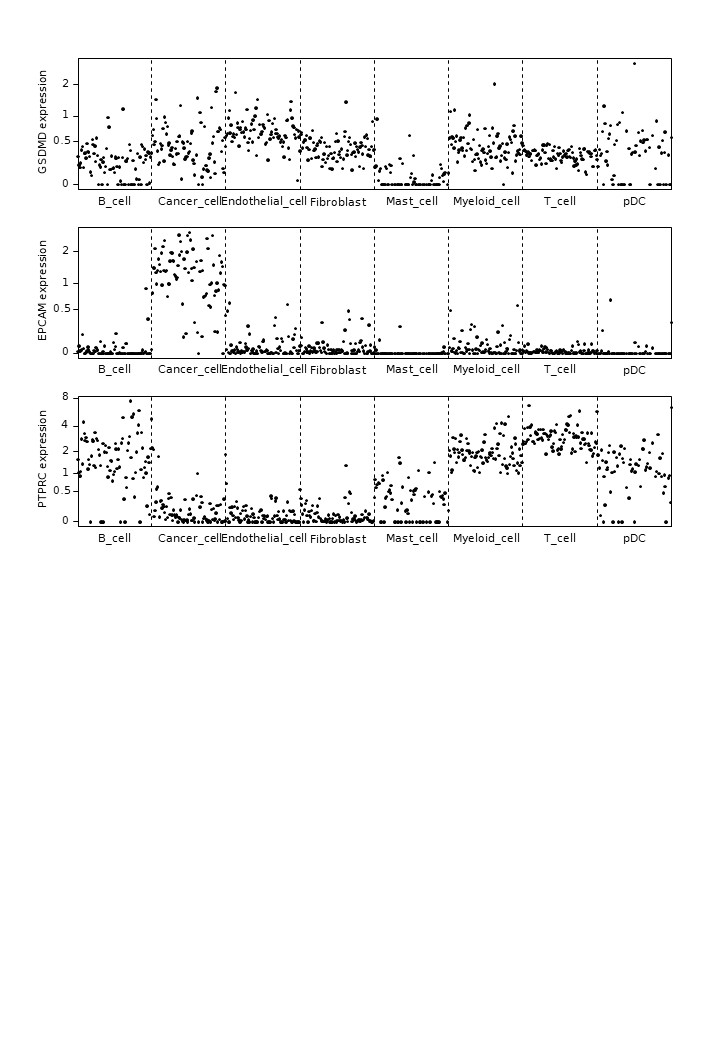

Supplement: Supplementary Figure 1 — Scatter plots showing the average expression of the genes GSDMD, EPCAM and PTPRC by tumor samples (individual dots) and cell types, based on scRNA dataset from Bassez et al. (2021) (breast cancer, n=84). [file Image1.jpeg]

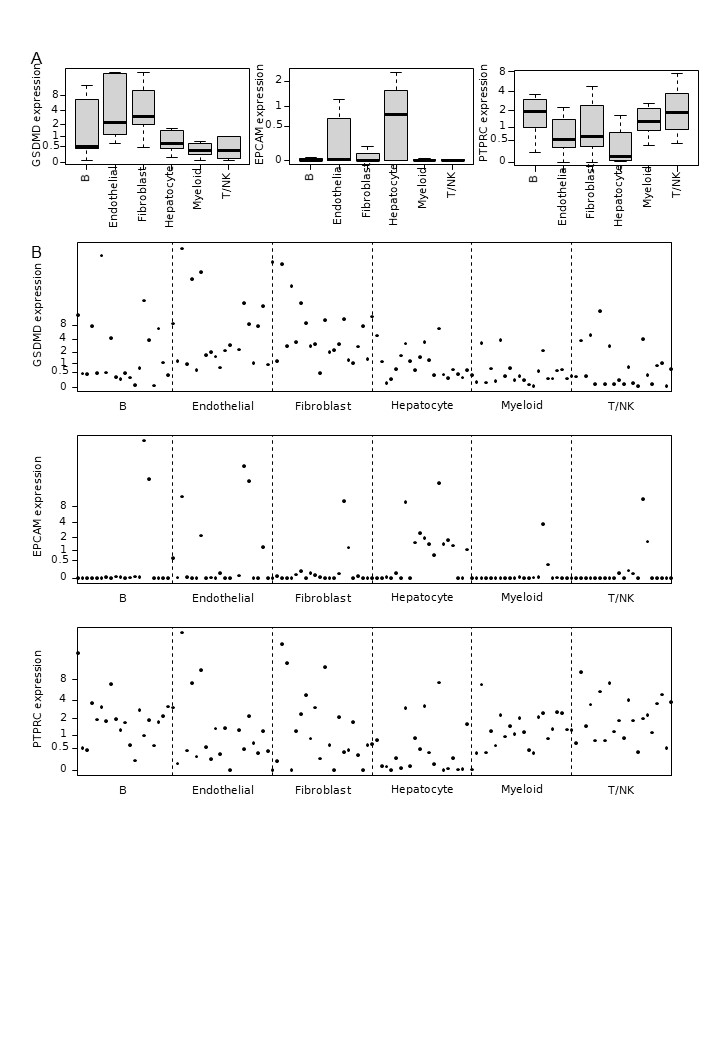

Supplement: Supplementary Figure 2 — (A) Boxplots comparing the distribution of per-sample, per-cell type average expression of the genes GSDMD, EPCAM (epithelial control) and PTPRC (immune cell control), in scRNA datasets from Lu et al. (2022) (hepatocellular carcinoma, n=21). (B) Scatter plots showing the average expression of the genes GSDMD, EPCAM and PTPRC by tumor samples (individual dots) and cell types, based on scRNA dataset from Lu et al. (2022) (hepatocellular carcinoma, n=21). [file Image2.jpeg]

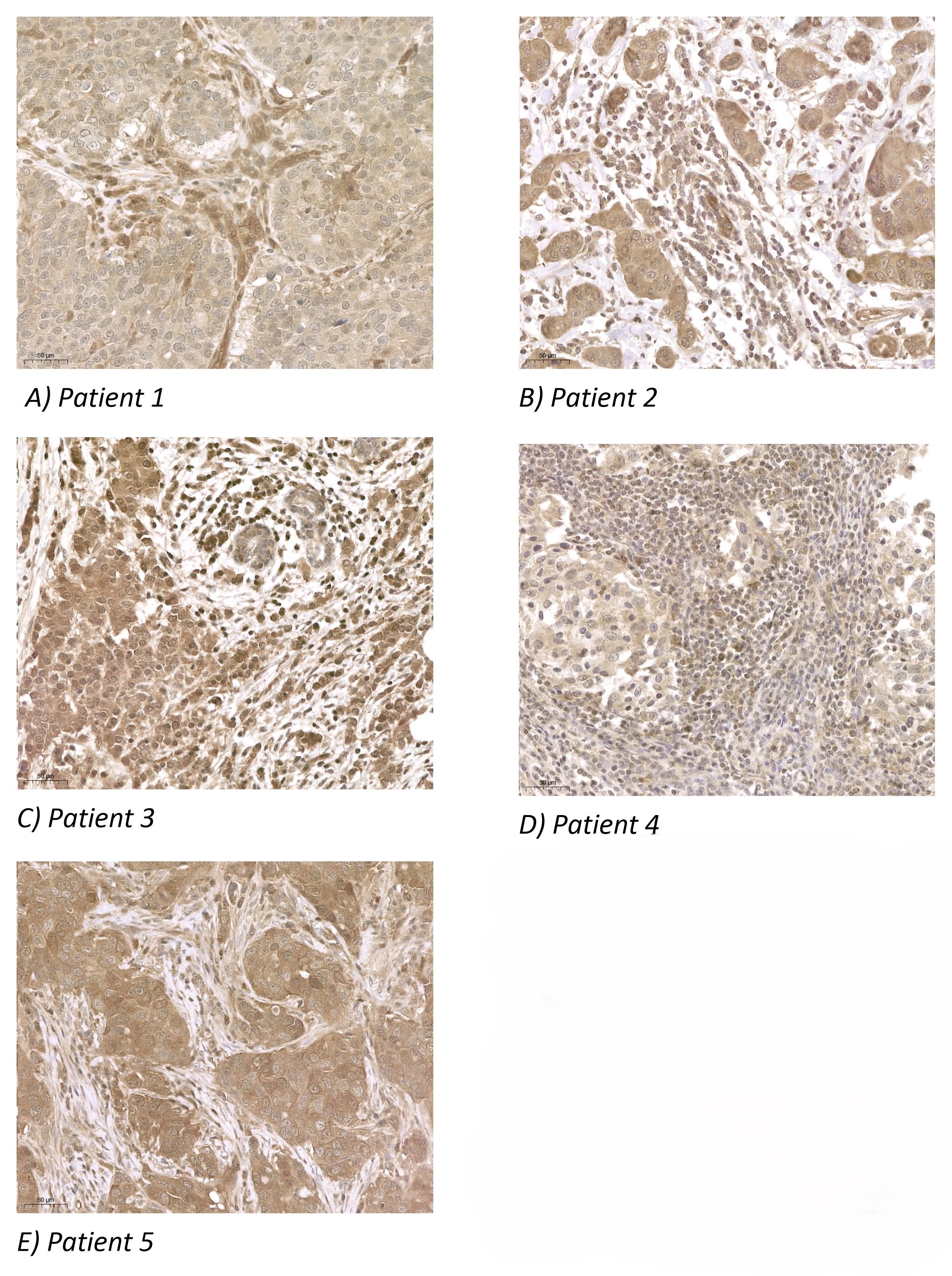

Supplement: Supplementary Figure 3 — (A–F) Human breast cancer samples stained for GSDMD (brown) by immunohistochemistry. [file Image3.jpeg]

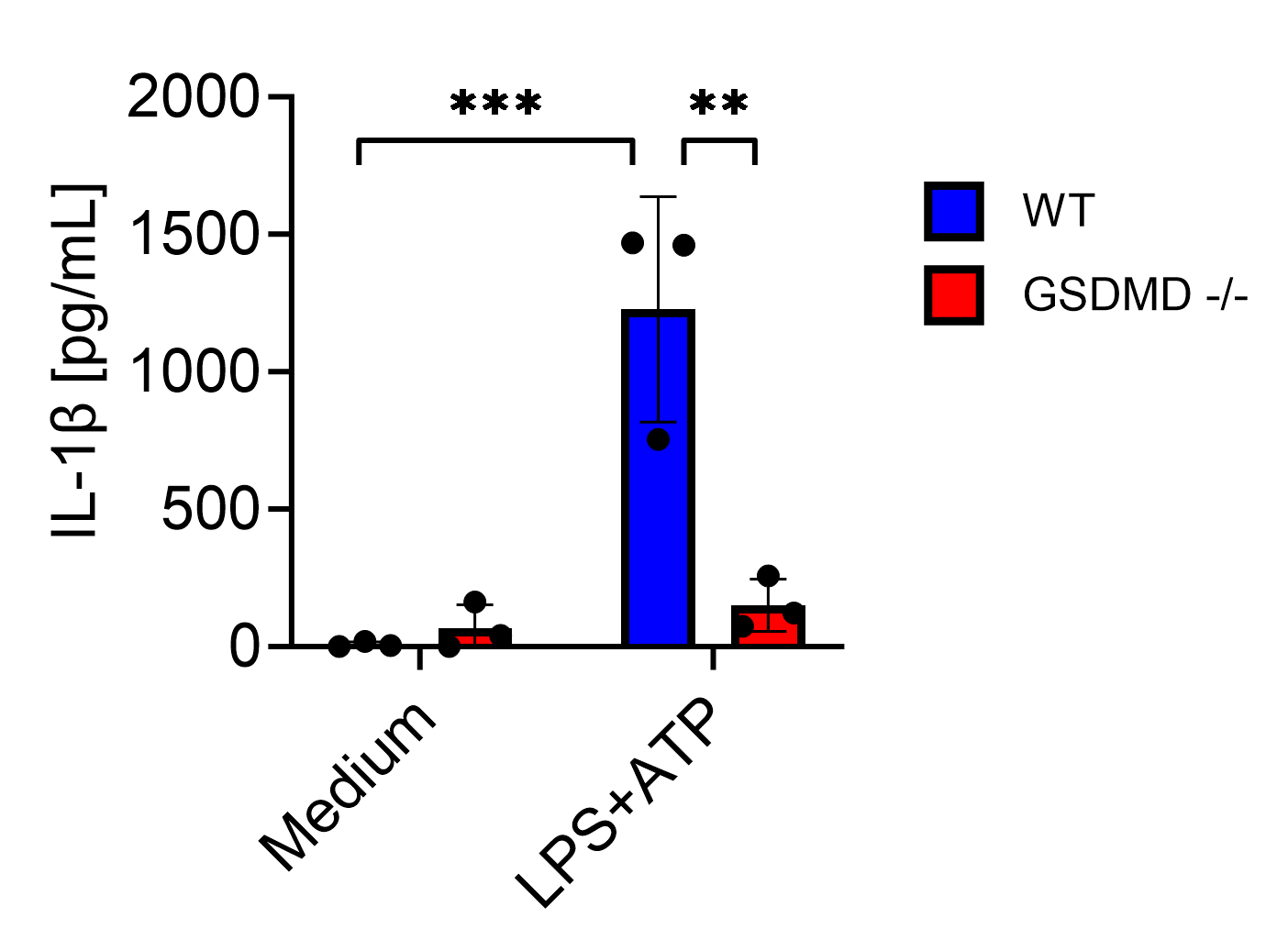

Supplement: Supplementary Figure 4 — IL-1β release from BMDM of WT (blue) and Gsdmd-/- (red) mice after 3h LPS (100ng/mL) followed by 1h ATP (5mM) treatment. [file Image4.tif]

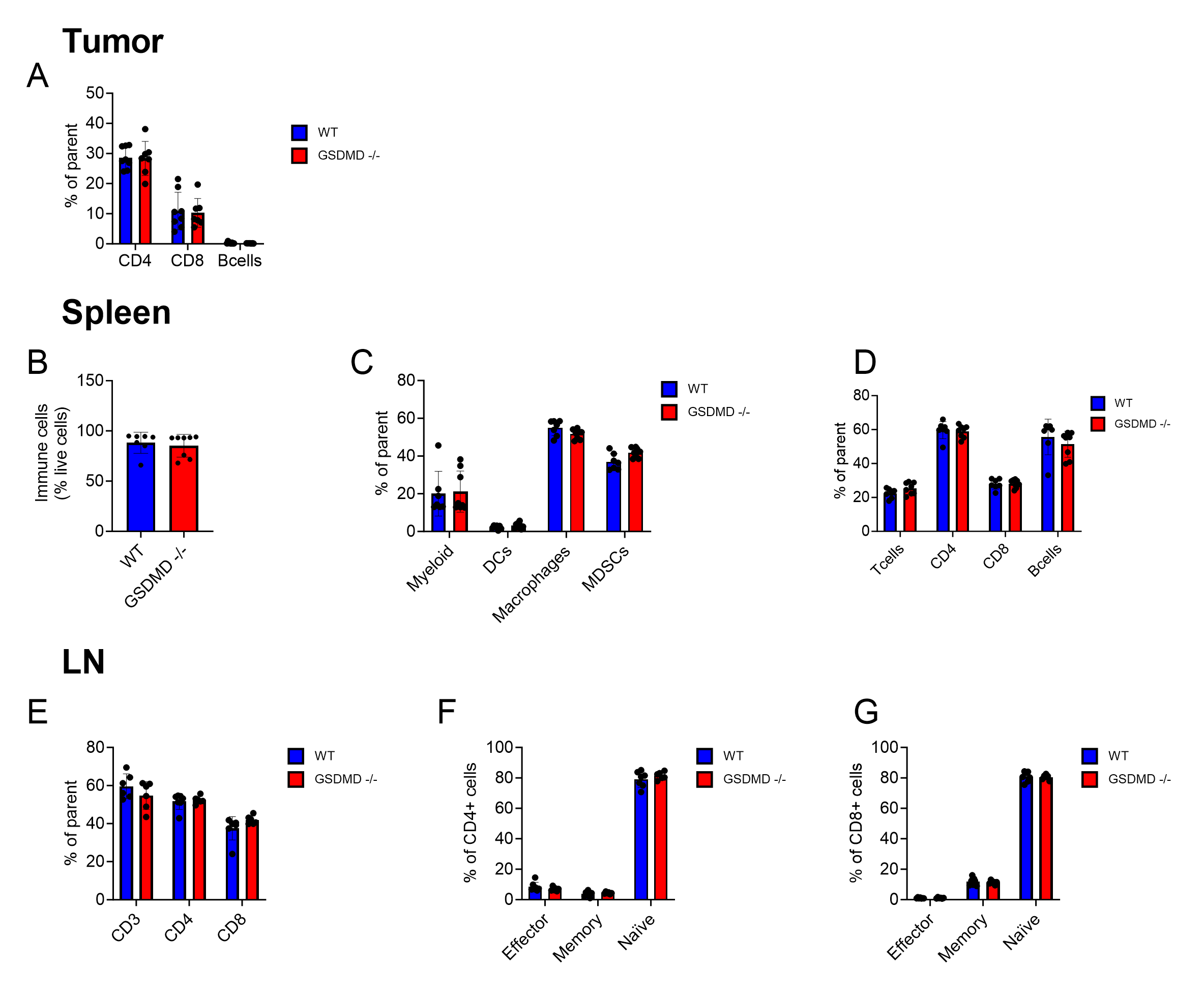

Supplement: Supplementary Figure 5 — (A–G) Flow cytometric analysis of immune cells in tumor (A), spleen (B–D) and lymph nodes (LN) (E–G) of EO771 tumor-bearing WT and Gsdmd-/- mice. (A) Percentages of CD4+ and CD8+ T cells (CD3 +) and B cells (CD19 +) in the tumor. Percentages of (B) CD45 + cells, (C) myeloid cells (CD11b +), DCs (CD11b +, CD11c +), macrophages (CD11b +, F4/80 +), and MDSCs (CD11b +, F4/80 +, GR1 +), and (D) total T cells (CD3+), CD4 + T cells, CD8 + T cells and B cells (D) in the spleen of EO771 tumor-bearing WT and Gsdmd-/- mice. (E) Percentages of total T cells (CD3+), CD4 + T cells and CD8 + T cells in the draining lymph nodes (LN) of EO771 tumor-bearing WT and Gsdmd-/- mice. Percentages of effector (CD44 +), memory (CD44 +, CD62L +) and naïve (CD62L +) CD4 + T cells (F) and CD8 + T cells (G) in draining lymph nodes of EO771 tumor-bearing WT and Gsdmd-/- mice. [file Image5.tif]

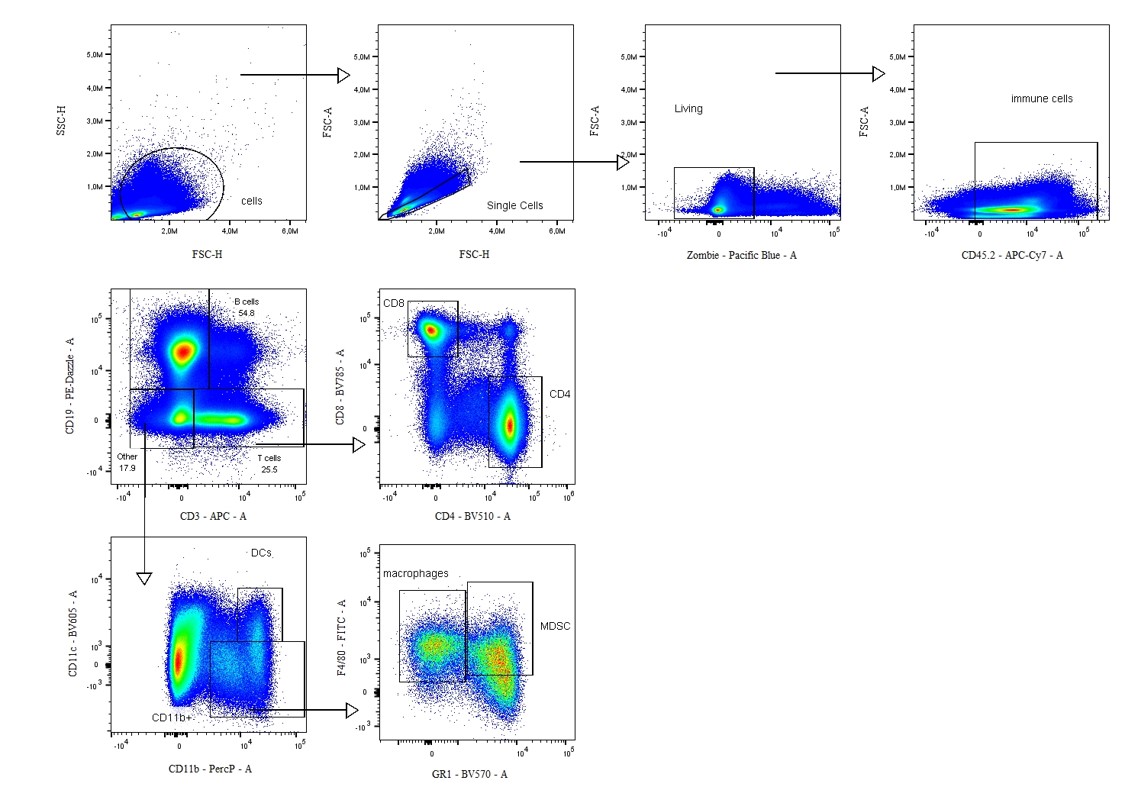

Supplement: Supplementary Figure 6 — Gating strategy for the analysis of EO771 and Hepa1-6 tumors and spleens of healthy or tumor bearing WT and Gsdmd-/- mice. The first gate was set to exclude cell debris. The second gate was used to select single cells. Then dead cells were excluded with the help of a viability marker. Using CD45, the fourth gate was used to select immune cells. Then different subsets of immune cells were defined as follows: CD19+, CD3+CD8+, CD3+CD4+, CD11b+CD11c+, CD11b+F4/80+GR1-, CD11b+F4/80-GR1+. [file Image6.jpeg]

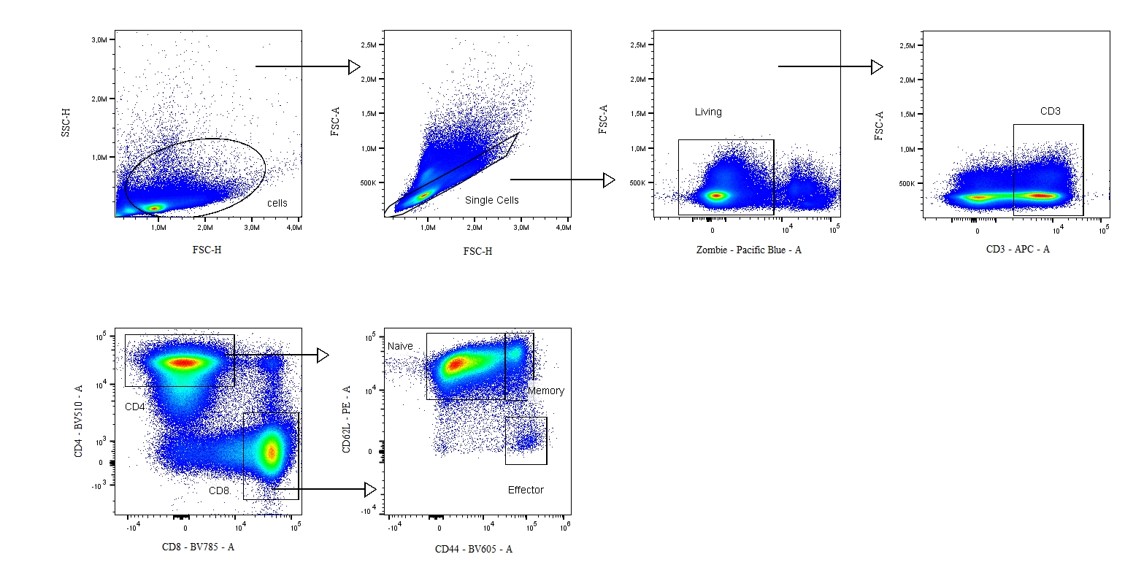

Supplement: Supplementary Figure 7 — Gating strategy for the analysis of LN of EO771 and Hepa1-6 tumor bearing WT and Gsdmd-/- mice. The first gate was set to exclude cell debris. The second gate was used to select single cells. Then dead cells were excluded with the help of a viability marker. Using CD45, the fourth gate was used to select immune cells. Then different subsets of immune cell subsets were defined as follows: CD3+CD8+, CD3+CD8+CD44+CD62L+, CD3+CD8+CD44- CD62L+, CD3+CD8+CD44+CD62L-, CD3+CD4+, CD3+CD4+CD44+CD62L+, CD3+CD4+ CD44-CD62L+, CD3+CD4+CD44+CD62L-. [file Image7.jpeg]

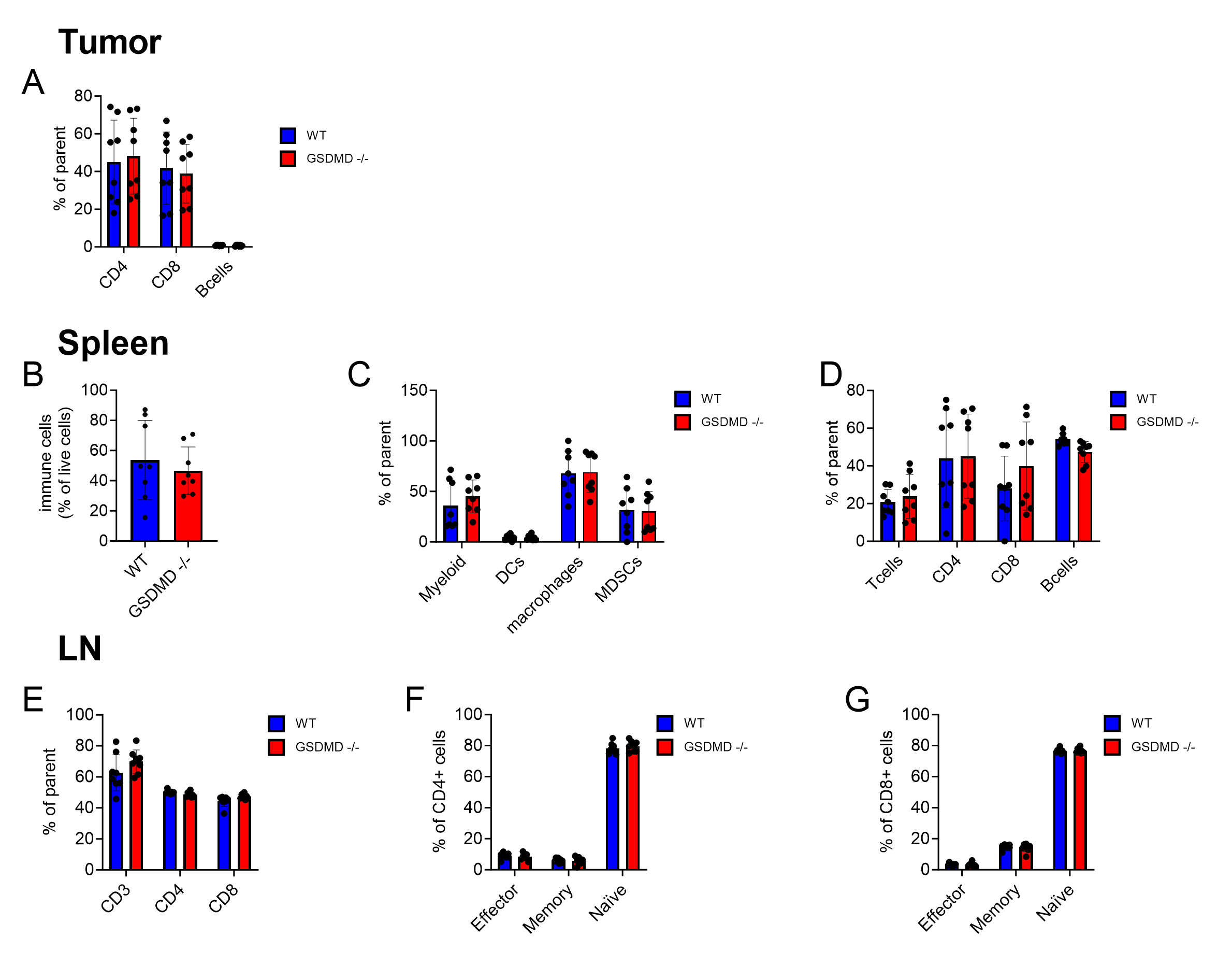

Supplement: Supplementary Figure 8 — (A–G) Flow cytometric analysis of immune cells in tumor (A), spleen (B–D) and lymph nodes (LN) (E–G) of Hepa1-6 tumor bearing WT and Gsdmd-/- mice. (A) Percentages of CD4+ and CD8+ T cells (CD3 +) and B cells (CD19 +) in the tumor. Percentages of (B) CD45 + cells, (C) myeloid cells (CD11b +), DCs (CD11b +, CD11c +), macrophages (CD11b +, F4/80 +), and MDSCs (CD11b +, F4/80 +, GR1 +), and (D) total T cells (CD3+), CD4 + T cells, CD8 + T cells and B cells (D) in the spleen of Hepa1-6 tumor-bearing WT and Gsdmd-/- mice. ((E) Percentages of total T cells (CD3+), CD4 + T cells and CD8 + T cells in the draining lymph nodes (LN) of Hepa1-6 tumor-bearing WT and Gsdmd-/- mice. Percentages of effector (CD44 +), memory (CD44 +, CD62L +) and naïve (CD62L +) CD4 + T cells (F) and CD8 + T cells (G) in draining lymph nodes of Hepa1-6 tumor-bearing WT and Gsdmd-/ mice. [file Image8.tif]

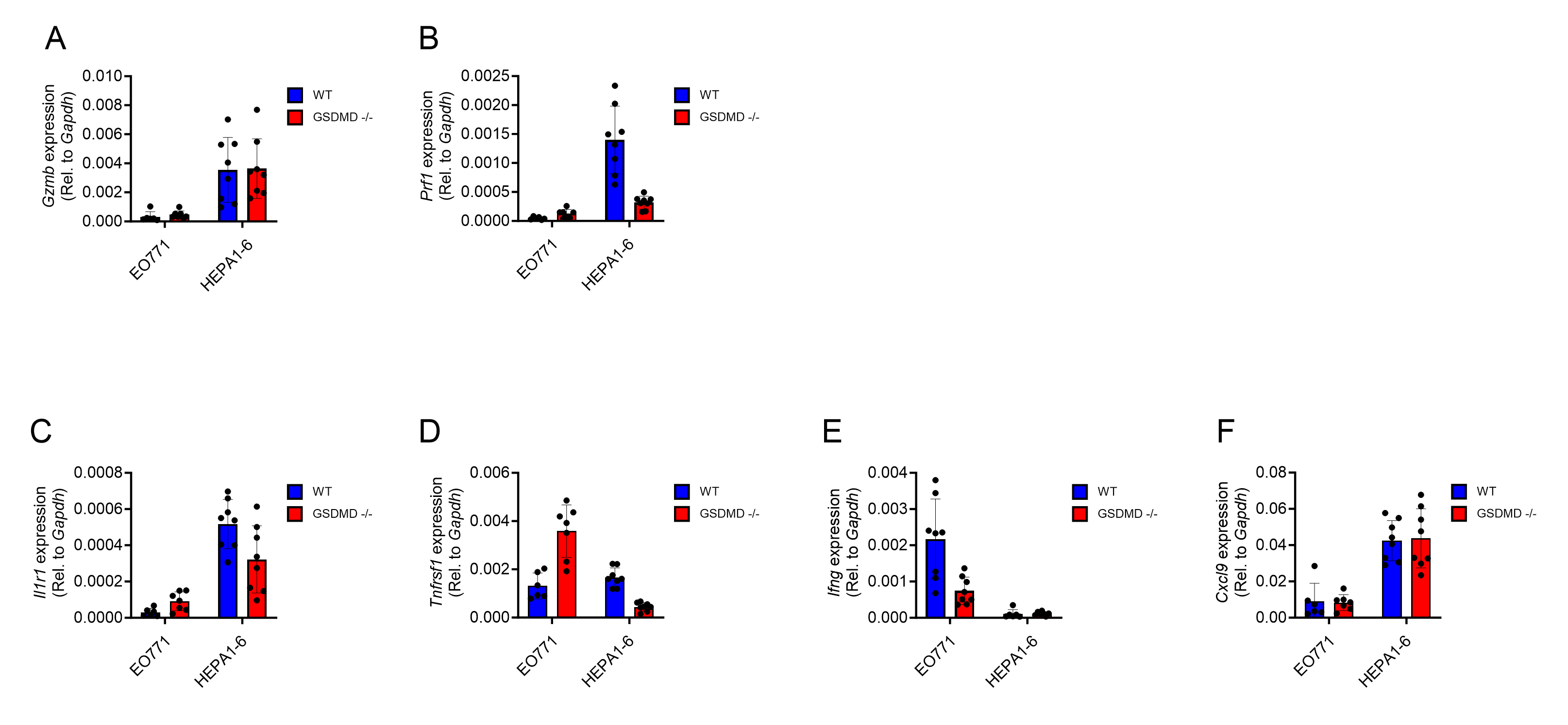

Supplement: Supplementary Figure 9 — (A–F) Gene expression of Gzmb (A), Prf1 (B), Il1r1 (C), Tnfrsf1 (D), Ifny (E) and Cxcl9 (F) in whole EO771 and Hepa1-6 tumor samples from WT and Gsdmd-/- mice. [file Image9.tif]

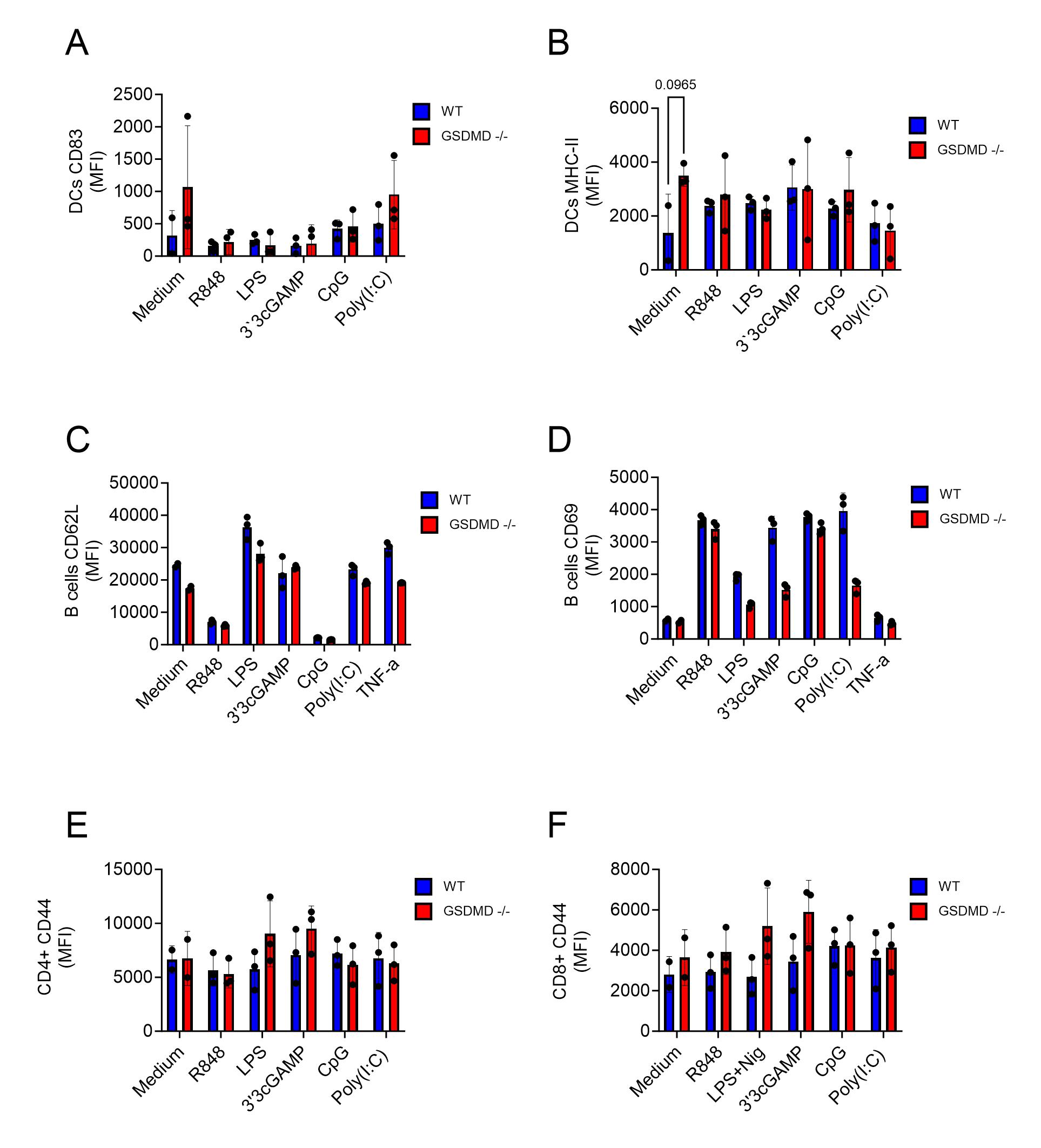

Supplement: Supplementary Figure 10 — (A–F) Bone marrow cells (A, B) and splenocytes (C–F) of WT and Gsdmd-/- mice were stimulated with R848 (0.1 μg/mL), LPS (0.1 μg/mL), 3’3’cGAMP (10 μg/mL), CpG (30 μg/mL) and Poly(I:C) (200 μg/mL). Cell surface levels of activation markers were measured 18 hours after stimulation. Cell surface CD83 (A) and MHC-II (B) levels on bone marrow DCs (CD11b+CD11c+). Cell surface CD62L (C) and CD69 (D) levels on spleen B cells (CD19+). Cell surface CD44 levels on spleen CD4 + T cells (CD3 +) (E) and CD8 + T cells (CD3 +) (F). [file Image10.jpeg]

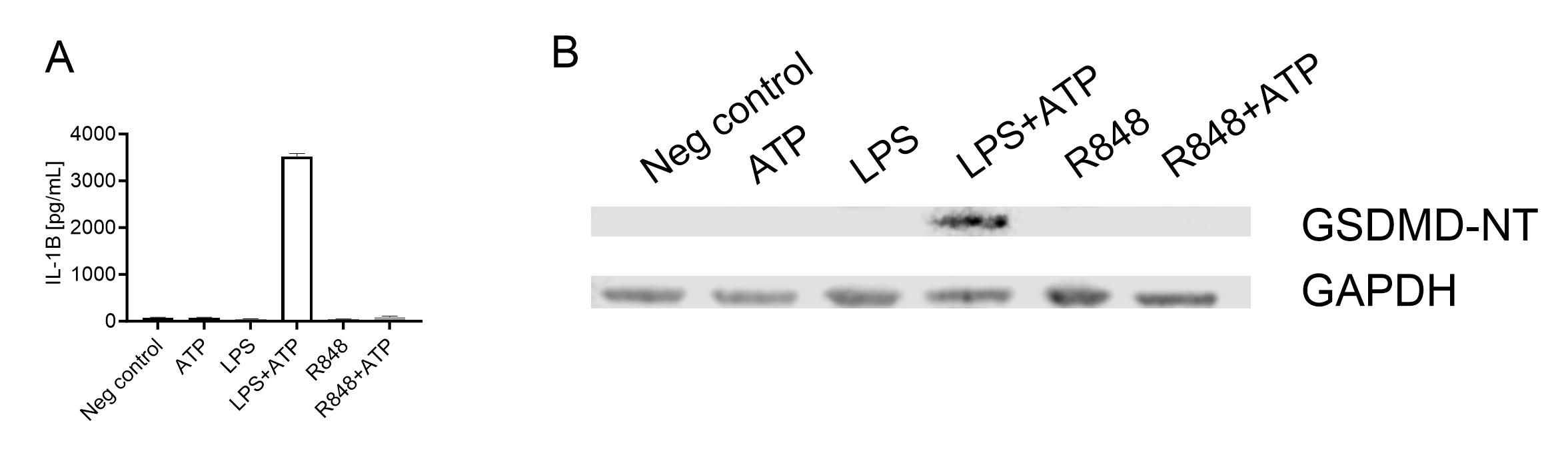

Supplement: Supplementary Figure 11 — IL-1β (A) GSDMD (B) levels from BMDM after 3h LPS (100ng/mL) or R848 (0.1 μg/mL) followed by 1h ATP (5mM) treatment. [file Image11.jpeg]

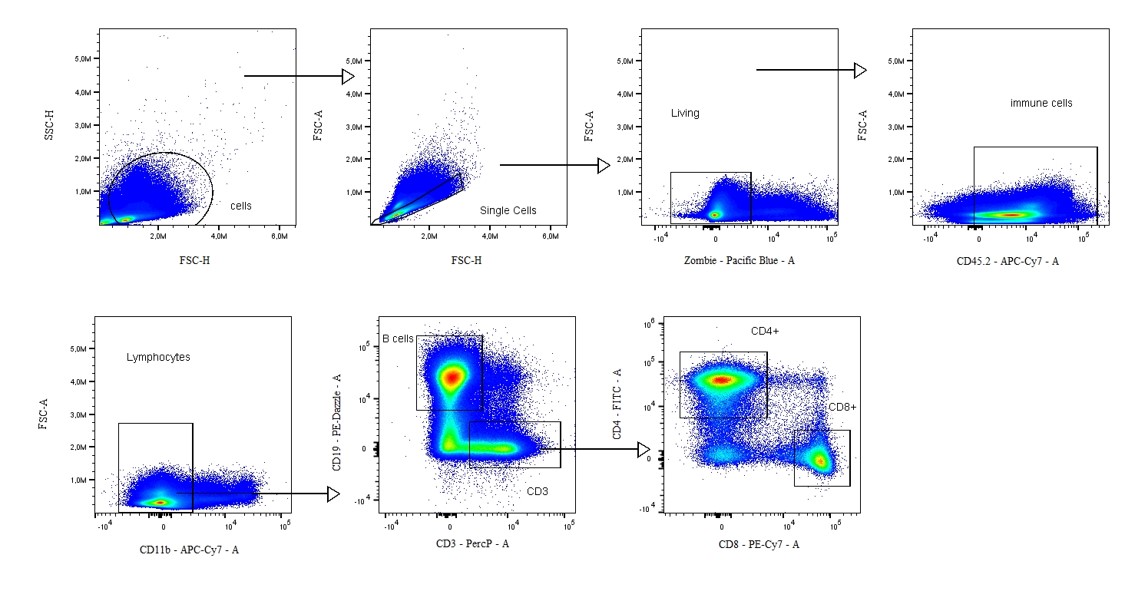

Supplement: Supplementary Figure 12 — Gating strategy for the analysis of stimulated splenocytes isolated from WT and Gsdmd-/- mice. The first gate was set to exclude cell debris. The second gate was used to select single cells. Then dead cells were excluded with the help of a viability marker. Using CD45, the fourth gate was used to select immune cells. Then different subsets of immune cell subsets were defined as follows: CD19+, CD3+CD8+, CD3+CD4+. [file Image12.jpeg]

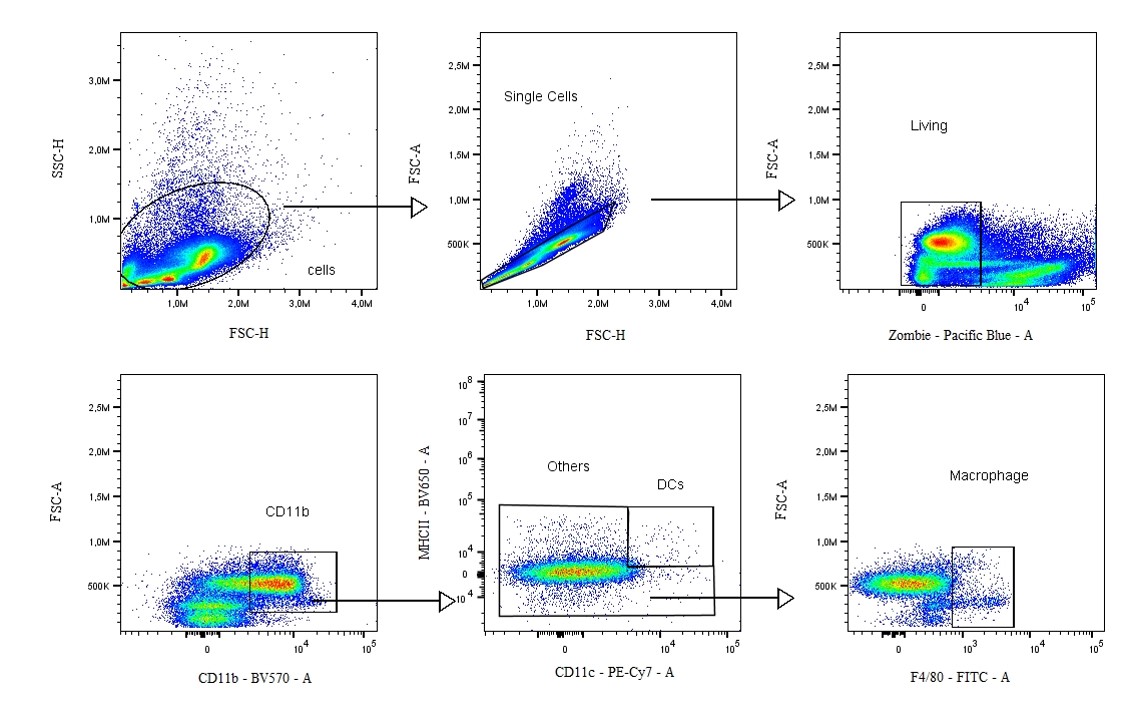

Supplement: Supplementary Figure 13 — Gating strategy for the analysis of stimulated bone marrow isolated from WT and Gsdmd-/- mice. The first gate was set to exclude cell debris. The second gate was used to select single cells. . Then dead cells were excluded with the help of a viability marker. Using CD45, the fourth gate was used to select immune cells. Then different subsets of immune cell subsets were defined as follows: CD11b+CD11c+ and CD11b+F4/80+. [file Image13.jpeg]

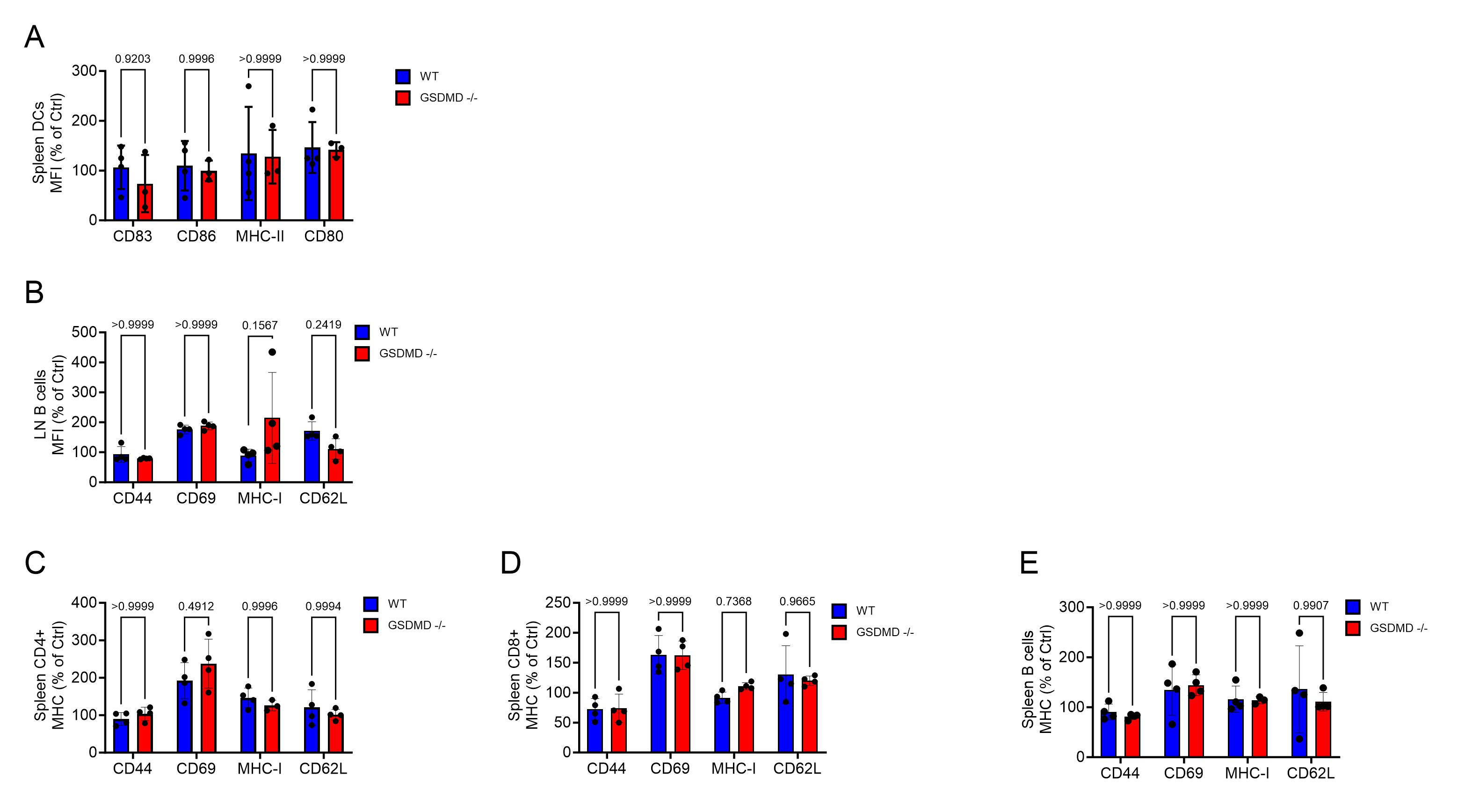

Supplement: Supplementary Figure 14 — (A–E) flow cytometric analysis of immune cells in lymph nodes (A) and spleen (B–E) 3H after R848 (10 μg, s.c.) or PBS injection in WT and Gsdmd-/- mice. (A) Cell surface CD44, CD69, MHC-I and CD62L levels in lymph node B cells (CD19+). (B) Cell surface CD83, CD86, MHCII and CD80 levels in spleenCD4+ T cells (CD3+) (C), CD8+ T cells (CD3+), (D) B cells (CD19+) and (E) DCs (CD11b+, CD11c+). [file Image14.jpeg]
